# β-aminobutyric acid primes lignin-associated resistance to *Penicillium digitatum* in postharvest lemon fruit involving ClMYB46L-mediated transcriptional activation

**DOI:** 10.3389/fpls.2026.1984112

**Published:** 2026-10-28

**Authors:** Chunhong Li, Kejiang Chen, Yijia Xia, Siling Li, Keda Jiang, Huaping Xu, Lichao Wen, Lu Rui, Kaituo Wang

**Affiliations:** 1 Institute of Fruit Function and Disease Management, Department of Public Health and Management, Chongqing Three Gorges Medical College, Chongqing, China; 2 College of Biology and Food Science, Chongqing Sanxia University of Science and Technology, Chongqing, China

**Keywords:** BABA, lemon fruit, lignin biosynthesis, MYB, *Penicillium digitatum*, postharvest resistance, transcriptome

## Abstract

Green mold caused by *Penicillium digitatum* severely limits the postharvest storage of lemon fruit. β-Aminobutyric acid (BABA) is known to enhance disease resistance in harvested fruit, but the transcriptional basis by which BABA reinforces defense responses in infected lemon peel remains insufficiently understood. Here, BABA pretreatment slowed the development of green mold in postharvest lemon fruit, as reflected by smaller lesions, reduced fungal biomass, and less infection-associated tissue damage. RNA-seq analysis showed that BABA did not merely intensify the overall response to *P. digitatum*, but reshaped a defined subset of pathogen-responsive genes. Among the enriched pathways, phenylpropanoid biosynthesis was particularly prominent, with lignin biosynthesis-related genes showing stronger induction in BABA-pretreated infected fruit than in fruit inoculated with the pathogen alone. Consistently, BABA pretreatment increased lignin accumulation and enhanced phloroglucinol-HCl staining around inoculation sites. A MYB46-like R2R3-MYB transcription factor, ClMYB46L, was identified as a candidate regulator associated with BABA-primed lignin biosynthesis. *ClMYB46L* expression was induced by *P. digitatum* challenge and was further increased in BABA-pretreated inoculated fruit. EMSA and Y1H assays showed that ClMYB46L binds SMRE-containing promoter fragments of lignin biosynthesis-related genes, including *Cl4CL*, *ClCCR*, *ClCCoAOMT*, *ClCOMT*, *ClCAD6* and *ClCAD9*. Dual-luciferase assays showed that ClMYB46L activates the corresponding full-length promoters. Transient overexpression of *ClMYB46L* in lemon fruit increased lignin deposition, up-regulated lignin biosynthetic genes, reduced fungal biomass and restricted lesion development. These findings suggest that BABA-enhanced resistance in postharvest lemon fruit is associated with ClMYB46L-mediated activation of lignin biosynthesis and local cell wall reinforcement.

## 1 Introduction

Lemon (*Citrus limon* (L.) Burm. f.) is an economically important citrus crop, with fruit used for fresh consumption and juice production, and peel serving as a source of bioactive compounds (Klimek-Szczykutowicz et al., 2020; Rafiq et al., 2018). However, lemon fruit are easily wounded during harvest, packing, and storage, creating entry sites for postharvest pathogens. Among postharvest citrus diseases, green mold caused by *Penicillium digitatum* is particularly damaging, leading to rapid lesion expansion, tissue maceration and substantial commercial losses (Bhatta, 2022). Although synthetic fungicides remain effective in many postharvest systems, fungicide-based control is increasingly limited by concerns over chemical residues, fungicide-resistant pathogen populations and consumer demand for safer preservation strategies (Papoutsis et al., 2019). Alternative approaches include natural antimicrobial compounds that act directly on the pathogen, for example by disrupting fungal membrane integrity and primary metabolism (Reymick et al., 2024). Therefore, enhancing the intrinsic defense capacity of harvested fruit has become an attractive approach for reducing postharvest decay while maintaining fruit quality (Romanazzi et al., 2016; Prusky and Romanazzi, 2023).

β-Aminobutyric acid (BABA) can prime plant immune responses, enabling host tissues to mount enhanced resistance upon pathogen attack (Thevenet et al., 2017). In a primed state, host tissues maintain relatively low basal defense activity before infection but show a stronger readiness to initiate defense responses after pathogen challenge (Conrath et al., 2006; Cohen et al., 2016). This priming mode is particularly relevant to harvested fruit, where excessive defense activation may increase metabolic costs and compromise postharvest quality (van Hulten et al., 2006). In postharvest systems, BABA has been reported to reduce fungal decay in grapefruit, grape and peach fruit, a protection generally associated with enhanced host defense responses after inoculation rather than direct inhibition of pathogen growth (Porat et al., 2003; Li et al., 2025). An earlier study on the same pathosystem showed that BABA pretreatment restricts green mold development in lemon through a two-phase redox response, in which early ROS-dependent defense signaling is retained while late-stage oxidative injury is limited (Li et al., 2026a). Whether BABA also strengthens structural defenses that physically constrain fungal spread, and how such a response is organized at the transcriptome level, has not been examined.

Cell wall reinforcement is closely related to the local defense of wounded fruit tissues (Bellincampi et al., 2014). Such local reinforcement is particularly relevant to *P. digitatum* infection, as this pathogen mainly enters citrus fruit through wounds and expands rapidly by promoting host tissue maceration (Marcet-Houben et al., 2012). A stronger cell wall barrier around infection sites may therefore restrict fungal penetration, delay lesion expansion and reduce tissue collapse (Lee et al., 2019). This structural defense is closely linked to phenylpropanoid metabolism, especially the lignin branch, which contributes to local cell wall strengthening while also generating defense-related phenolic compounds (Dixon et al., 2002; Miedes et al., 2014). In citrus fruit, several studies have linked phenylpropanoid activation with resistance to *P. digitatum*. For example, PAL and peroxidase activities show spatial changes around infection sites during the citrus–*P. digitatum* interaction, suggesting that local phenylpropanoid responses are associated with host defense (Ballester et al., 2006). Transcriptomic and metabolic analyses have further shown that citrus fruit with induced resistance often display coordinated up-regulation of phenylpropanoid metabolism genes together with higher levels of defense-related phenylpropanoid compounds (Ballester et al., 2011, 2013). More recently, lignin biosynthesis has also been implicated in transcription factor-mediated citrus resistance to *P. digitatum* (Chen et al., 2023). These observations point to lignification as a potentially important defense output in citrus peel, but whether BABA enhances postharvest lemon resistance through transcriptome-level activation of lignin biosynthesis remains unclear.

The transcriptional regulation of lignin biosynthesis is largely mediated by MYB transcription factors (Zhong and Ye, 2009). In the secondary cell wall network, R2R3-MYB proteins occupy key positions and directly or indirectly regulate many lignin pathway genes (Zhong and Ye, 2012; Xiao et al., 2021). In *Arabidopsis*, MYB46 and MYB83 function as major regulators of secondary wall formation and coordinate the expression of genes required for cellulose, hemicellulose and lignin biosynthesis (McCarthy et al., 2009; Ko et al., 2014). Other MYB members, such as MYB58 and MYB63, act more directly on the lignin branch and activate lignin biosynthetic genes, thereby promoting lignin deposition (Zhou et al., 2009). These conserved regulators provide useful clues for identifying lignin-associated MYB regulators in lemon fruit. However, compared with model plants, much less is known about whether R2R3-MYB regulators participate in inducible lignification during postharvest disease resistance, especially in citrus fruit. Identifying a BABA-responsive R2R3-MYB regulator and clarifying its direct targets may therefore help explain how BABA-mediated transcriptional reprogramming is converted into lignin accumulation and enhanced resistance in lemon fruit.

Here, we explored the role of BABA in shaping the defense response of postharvest lemon fruit to *P. digitatum*, with a focus on lignin-related cell wall reinforcement. After confirming the protective effect of BABA against green mold, we used transcriptome profiling to examine how BABA pretreatment altered the response of infected peel tissues. The transcriptomic data highlighted phenylpropanoid and lignin biosynthesis as major BABA-responsive pathways, suggesting that enhanced lignification may contribute to the restricted development of green mold. We therefore examined lignin accumulation and focused on ClMYB46L, a MYB46-like R2R3-MYB transcription factor, to test whether this regulator links BABA-associated transcriptional changes with lignin biosynthesis and enhanced resistance in lemon fruit.

## 2 Materials and methods

### 2.1 Biological materials

*P. digitatum* from our laboratory collection was propagated, and the conidial suspension prepared and adjusted to 1.0 × 106 conidia mL-1, as described previously (Li et al., 2026a).

Commercially mature ‘Eureka’ lemons (*Citrus limon* (L.) Burm. f.) were collected from a uniformly managed orchard and brought to the lab within 2 h at approximately 20 °C in autumn 2025. Fruit with comparable external quality and without visible defects, pest injury, or disease symptoms were screened for the experiment and allowed to stand at 20 °C before treatment.

*Nicotiana benthamiana* plants used for leaf infiltration were grown as described previously (Wang et al., 2025), and non-flowering plants of approximately two months were used.

### 2.2 BABA pretreatment and *P. digitatum* challenge

After pre-cooling, the fruit surface was disinfected with 75% (v/v) ethanol and allowed to dry in air. Lemons were then randomly allocated to four groups: Control, BABA, Inoculation, and BABA+INO, with 360 fruit in each group. Two wounds were made at opposite sides of the equatorial region according to a previous method (Li et al., 2025). Control wounds received sterile water, BABA wounds received 20 mM β-aminobutyric acid, and Inoculation wounds received a *P. digitatum* conidial suspension, with 20 μL applied per wound. For the combined treatment, BABA was applied first and the pathogen suspension was added 6 h later. The selected BABA dose and pretreatment interval were chosen according to previous postharvest studies (Porat et al., 2003; Li et al., 2026a).

After each application, fruit were allowed to dry in air. Following the final treatment, fruit were placed in 0.06-mm polyethylene bags containing four fruit each, and maintained at 20 °C under 80–90% relative humidity. Samples of peel tissue (60–80 g) were taken at 3-d intervals from tissue located about 2 mm away from the lesion boundary and then frozen immediately in liquid nitrogen before storage at −80 °C. In fruit without pathogen inoculation, the corresponding wounded region was sampled. For disease assessment, 60 fruit from each treatment were kept intact and monitored during storage. For biochemical and molecular analyses, three biological replicates were included at each sampling point, and each replicate consisted of pooled peel tissue from 12 lemons, corresponding to 24 wounds. Similar results were obtained in two independent experiments.

### 2.3 Evaluation of disease development

Disease progression was assessed at 3-d intervals during storage. Lesion diameter at the two inoculation wounds on each fruit was recorded, and their average was used for analysis. The relative abundance of *P. digitatum* from inoculated peel samples was determined by DNA-based qPCR according to previously reported methods (Li et al., 2026a; Zhang et al., 2020a).

### 2.4 Trypan blue staining

Cell death and fungal colonization in lemon peel tissues were visualized by trypan blue staining with minor modifications to a previously reported method (Cai et al., 2020). In brief, infected lemon peel tissues were incubated in a staining mixture consisting of 0.025% (w/v) trypan blue, 25% (w/w) phenol, 25% (v/v) lactic acid, and 25% (v/v) glycerol, followed by destaining with saturated chloral hydrate. After clearing, samples were observed using a stereomicroscope (Leica EZ4D, Germany) to evaluate host cell death and fungal growth.

### 2.5 RNA-seq library preparation and data analysis

Total RNA was isolated from lemon peel tissues, and its integrity was evaluated with an Agilent 2100 Bioanalyzer. Each treatment included three biological replicates. After quality control, mRNA was purified with Oligo(dT) magnetic beads and randomly fragmented, followed by cDNA library construction according to the standard protocol provided by Novogene. The constructed libraries were checked using Qubit 2.0 and an Agilent 2100 Bioanalyzer, and libraries that passed quality control were subjected to paired-end sequencing. Raw sequencing data were filtered by removing reads with adapters, reads containing ambiguous bases (N), and low-quality reads to obtain clean reads (Wang et al., 2009). The *Citrus limon* reference genome and annotation files (cncb_citrus_limon_Citrus_limon_Lemon_GWHCBFU00000000) were downloaded from the Genome Warehouse (GWH) of the National Genomics Data Center, China National Center for Bioinformation. Clean reads were mapped to the reference genome with HISAT2 v2.2.1 (Kim et al., 2019), and transcript abundance was calculated with featureCounts v2.0.6 (Liao et al., 2014). Differentially expressed genes (DEGs) were screened with DESeq2 v1.42.0 using thresholds of |log2(fold change)| ≥ 1 and adjusted *P* < 0.05 as the cutoff criteria (Love et al., 2014). GO and KEGG enrichment analyses were conducted with clusterProfiler v4.8.1 (Wu et al., 2021). Heatmaps of selected lignin biosynthesis-related DEGs were generated using TBtools based on TPM values from the three biological replicates of each treatment.

### 2.6 RT-qPCR analysis

cDNA was prepared from total RNA using a commercial reverse transcription kit (Takara, China). RT-qPCR was performed using SYBR Green reagent (Vazyme, China) on an ABI 7500 Fast Real-Time PCR System (Applied Biosystems) with three technical replicates per sample. Amplification conditions followed Li et al. (2025). Primers for target genes were designed based on gene sequences retrieved from the Genome Warehouse (GWH) reference annotation used for RNA-seq analysis, whereas the citrus reference genes *EGIDH* and *GAPC2* were selected according to their NCBI reference sequences. All primer sequences are provided in **Supplementary Table 1**. Relative expression levels were determined by the 2^-ΔΔCt^ method (Livak and Schmittgen, 2001) with *EGIDH* and *GAPC2* as internal controls. For data normalization, the control sample for each sampling point was assigned a value of 1.

### 2.7 Lignin quantification

Lignin levels in lemon peel were quantified using the acetyl bromide assay (Fukushima and Hatfield, 2001). Peel samples adjacent to the wound sites were collected from different treatments, frozen in liquid nitrogen, and milled into a fine powder. Approximately 0.10 g of powdered tissue was extracted with 1 mL of 95% ethanol and centrifuged to remove soluble phenolics and pigments. The residue was rinsed sequentially with 95% ethanol and n-hexane, and then dried. The dried residue was reacted with 25% acetyl bromide in glacial acetic acid at 70 °C for 0.5 h. After the samples cooled to room temperature, the reaction was stopped using 2 mol L^−^¹ NaOH, and the solution volume was adjusted with glacial acetic acid. After centrifugation, supernatant absorbance was recorded at 280 nm. Lignin concentration was estimated using a standard curve and expressed as mg g^−^¹ FW.

### 2.8 Histochemical staining of lignin

Lignin deposition in lemon peel was visualized using the phloroglucinol-HCl staining method (Blaschek et al., 2020). Peel samples adjacent to the wound sites were cut into approximately 1–2 mm thick longitudinal slices and placed in glass Petri dishes. The slices were fully immersed in freshly prepared 1% (w/v) phloroglucinol in ethanol for 3–5 min at room temperature under dark conditions. The dye solution was then discarded without rinsing, and the samples were immediately immersed in concentrated HCl (approximately 37%) for 1–2 min. After staining, the slices were quickly removed and photographed under a stereomicroscope. Lignified regions appeared red, reddish-purple, or reddish-brown, and staining intensity was used to compare relative lignin deposition among treatments.

### 2.9 Screening and phylogenetic analysis of lemon R2R3-MYB candidates associated with lignin regulation

To identify lemon MYB transcription factors potentially involved in lignin biosynthesis, MYB-related genes were first screened from the *Citrus limon* cv. Eureka v1.0 annotation dataset. Genes annotated as MYB transcription factors and containing the MYB DNA-binding domain (PF00249) were retained, while MYB-related, MYB3R-like, incomplete, abnormal, or redundant entries were removed. To reduce redundancy caused by closely related or fragmented gene models, candidates with identical or highly similar functional annotations were further checked, and representative non-redundant sequences were retained based on annotation reliability and sequence completeness.

Coding sequences for the retained lemon MYB candidates were extracted from the *C. limon* cv. Eureka v1.0 CDS dataset available in the Genome Warehouse (GWH) and translated into protein sequences. *Arabidopsis* MYB proteins associated with lignin biosynthesis and its regulation, including AtMYB46, AtMYB83, AtMYB58, AtMYB63, and AtMYB85 (Zhou et al., 2009; Xiao et al., 2021), were included as reference sequences. The lemon candidate proteins and *Arabidopsis* reference proteins were aligned using MEGA v5.02. A neighbor-joining tree was generated based on the full-length protein alignment with Poisson correction and pairwise deletion for gaps or missing data, and node support was tested using 1,000 bootstrap replicates. Based on the phylogenetic topology, evm_TU_ctg99_12 showed the closest relationship with *Arabidopsis* AtMYB46 and was therefore designated as ClMYB46L. This gene was selected for subsequent expression analysis and functional validation.

### 2.10 Electrophoretic mobility shift assay

The complete coding region of ClMYB46L was cloned into pGEX-4T-1 for GST-fusion protein expression in *Escherichia coli* Rosetta-gami (DE3) pLysS. Recombinant protein was induced by IPTG and purified using Glutathione Agarose resin.

Promoter probes were prepared from *Cl4CL*, *ClCCR*, *ClCCoAOMT*, *ClCOMT*, *ClCAD6*, and *ClCAD9*. Candidate MYB-binding sites were predicted by scanning the 2,000 bp upstream promoter sequences for the secondary wall MYB-responsive element (SMRE) consensus motif, ACC(A/T)A(A/C)(C/T), with both forward and reverse-complement strands considered. Where multiple SMREs were present, one representative site per promoter was selected for probe design. Wild-type probes containing the predicted SMRE-like motifs were labeled with biotin, and corresponding mutant probes carrying substitutions within the core motif served as mutant controls. Binding reactions were conducted with a chemiluminescent EMSA kit following the method reported by Ji et al. (2021). For competition analysis, unlabeled wild-type or mutant probes were added at a 50-fold molar excess. The reaction mixtures were resolved on native polyacrylamide gels, blotted onto nylon membranes, and visualized with a ChemiDoc MP imaging system (Bio-Rad).

### 2.11 Yeast one-hybrid assay

Promoter fragments from Cl4CL, ClCCR, ClCCoAOMT, ClCOMT, ClCAD6, and ClCAD9 containing predicted SMRE-like motifs were selected for Y1H analysis. Three repeated copies of each promoter element containing the candidate MYB-binding site were synthesized and cloned into pAbAi to generate bait constructs. After linearization, the bait vectors were introduced into *Saccharomyces cerevisiae* Y1HGold, and transformants were screened on SD/-Ura medium following Wang et al. (2025). Before interaction testing, the appropriate aureobasidin A (AbA) concentration for each bait strain was determined.

The full-length ClMYB46L CDS was inserted into pGADT7 to generate the prey construct. Each bait strain was transformed with either ClMYB46L-pGADT7 or the empty pGADT7 vector. Protein–DNA interaction was evaluated based on yeast growth on selective medium supplemented with AbA. The p53-AbAi/pGADT7–53 combination served as the positive control.

### 2.12 Dual-luciferase reporter assay

The promoter regions of *Cl4CL*, *ClCCR*, *ClCCoAOMT*, *ClCOMT*, *ClCAD6*, and *ClCAD9* were cloned into pGreenII 0800-LUC ahead of the firefly luciferase (LUC) coding region to obtain reporter constructs. The ClMYB46L coding sequence was inserted into pGreenII 62-SK to serve as the effector. All plasmids were introduced into *Agrobacterium tumefaciens* GV3101 (pSoup) following Li et al. (2026a).

Agrobacterial cells were resuspended in infiltration buffer containing 10 mM MgCl_2_, 10 mM MES (pH 5.6), and 100 μM acetosyringone, and the suspension was adjusted to OD_600_ of 0.5. Reporter and effector cultures were combined and co-infiltrated into fully expanded leaves of *N. benthamiana*. After 60–70 h, LUC and Renilla luciferase (REN) activities were detected with a GloMax Discover microplate reader (Promega, USA). Relative promoter activity was calculated as the LUC/REN ratio and normalized against the empty-effector control. Each treatment included at least twelve independent biological replicates.

### 2.13 Transient overexpression in lemon fruit

Transient expression of *ClMYB46L* in lemon fruit was achieved by *Agrobacterium tumefaciens*-mediated infiltration following Li et al. (2017) and Li et al. (2026a), incorporating modifications from a transformation system optimized for fruit tissues (Li et al., 2026b). The full-length *ClMYB46L* coding region was inserted into pGreenII 62-SK driven by the CaMV 35S promoter, and the resulting vector was introduced into *A. tumefaciens* GV3101. The empty pGreenII 62-SK vector served as the control. Agrobacterial suspensions were prepared to an OD_600_ of 0.8 and infiltrated at two opposite sites on each lemon. Lemons receiving the empty vector were used as EV controls, whereas fruit infiltrated with 35S::*ClMYB46L* were assigned to four independent transformation batches, designated OE-1, OE-2, OE-3, and OE-4.

At 2 d after infiltration, fruit were subjected to pathogen inoculation with or without BABA pretreatment. For the BABA+INO treatment, 20 μL of 20 mM BABA was applied to the infiltrated site 24 h before inoculation with 20 μL of *P. digitatum* conidial suspension (1.0 × 10^6^ conidia mL^-1^). A 24 h BABA pretreatment interval was used in this transient overexpression assay to accommodate the additional Agrobacterium infiltration step and to ensure sufficient BABA responsiveness before pathogen challenge. Peel tissues adjacent to the treated area were collected at 2 dpi. Lesion diameter, relative fungal biomass, lignin content, phloroglucinol-HCl staining and trypan blue staining were determined in inoculated (INO) fruit, whereas expression of lignin biosynthesis-related genes was analyzed under both INO and INO+BABA conditions. Each treatment included at least 20 fruits, and three biological replicates were performed.

### 2.14 Statistical analysis

Values are presented as means ± SD. All statistical tests were conducted in GraphPad Prism Version 8.0.1 (GraphPad Software, San Diego, CA, USA). For time-course experiments, two-way ANOVA was applied to evaluate treatment effects, time effects, and treatment × time interactions, with multiple testing corrected using the two-stage Benjamini-Krieger-Yekutieli procedure when appropriate. Single-factor experiments were analyzed by one-way ANOVA followed by Tukey’s multiple comparisons test. Statistical significance was defined as *P* < 0.05 or adjusted *P* < 0.05, as applicable. For time-course data, asterisks indicate the significance of the treatment effect (column factor): *P* < 0.05 (*), *P* < 0.01 (**), *P* < 0.001 (***), and *P* < 0.0001 (****).

## 3 Results

### 3.1 BABA pretreatment mitigates *P. digitatum*-induced decay in postharvest lemon fruit

Lemon fruit infected with *P. digitatum* displayed typical green mold symptoms, and the affected area enlarged progressively during storage, especially from 9 dpi onward (**Figure 1A**). In contrast, fruit receiving BABA before pathogen inoculation exhibited a slower development of decay symptoms, with smaller necrotic areas and less severe tissue collapse throughout the observation period. Non-inoculated fruit, including both Control and BABA-treated samples, generally maintained an intact appearance, although a few slight age-related spots appeared at the later stages.

**Figure 1:**
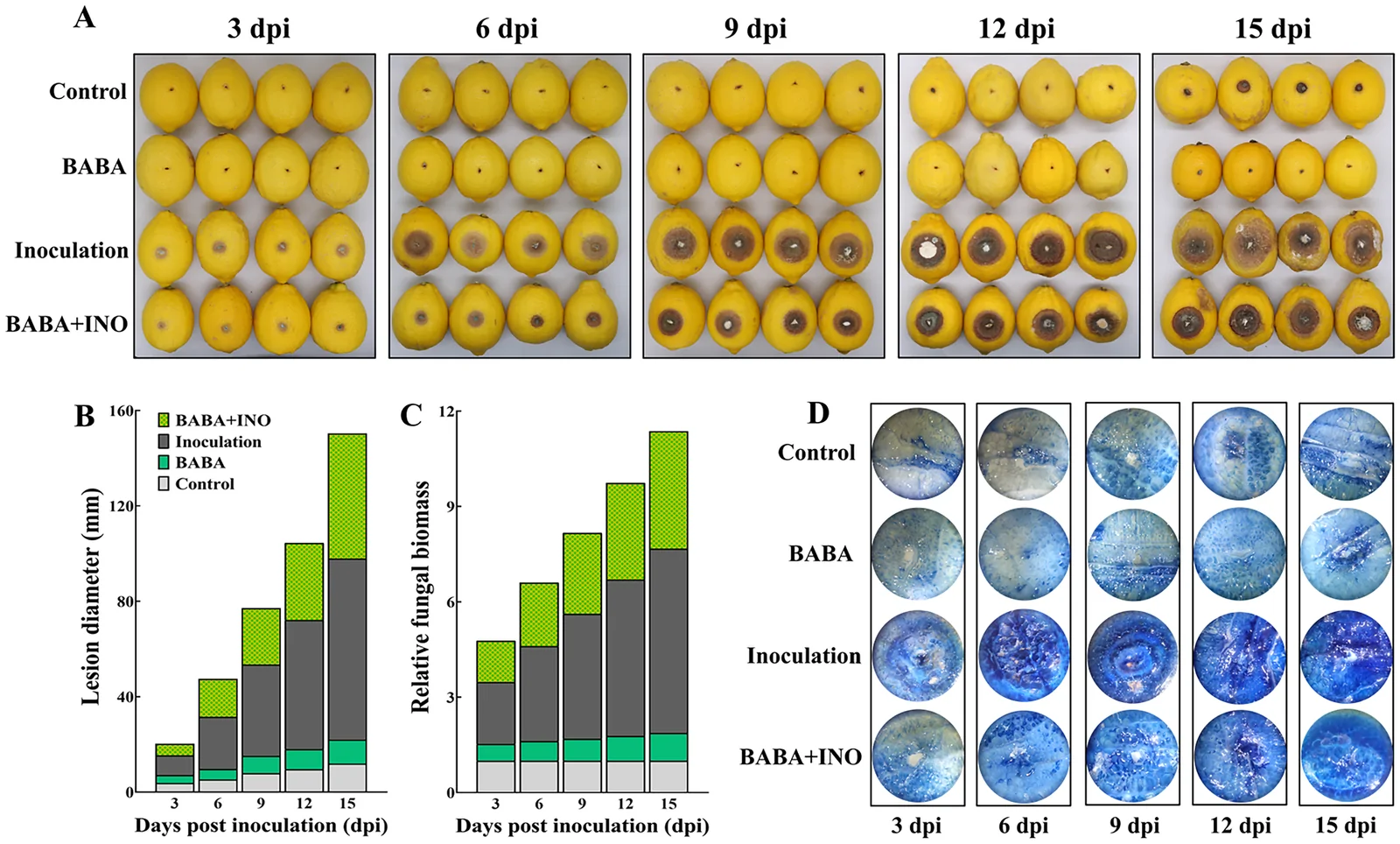
BABA delays disease progression and reduces *Penicillium digitatum* colonization in postharvest lemon fruit. **(A)** Representative disease development in lemon fruit receiving water, BABA, *P. digitatum*, or BABA followed by *P. digitatum* inoculation. Images were recorded at 3, 6, 9, 12 and 15 dpi. **(B)** Lesion expansion in lemon fruit during storage. **(C)** Relative fungal biomass in peel tissues collected from different treatment groups. **(D)** Trypan blue-stained peel tissues showing pathogen-associated staining and local tissue damage around inoculation sites.

Consistent with our previous report on this system (Li et al., 2026a), lesion diameter increased continuously in inoculated fruit, whereas BABA pretreatment markedly restricted lesion enlargement after pathogen challenge (*P* = 0.0173, 0.0074, 0.012, 0.0084 and 0.0065 at 3, 6, 9, 12 and 15 dpi, respectively) (**Figure 1B**). The DNA-based qPCR assay showed a similar trend in fungal biomass. *P. digitatum* accumulated gradually in inoculated fruit as storage progressed, but this increase was noticeably limited by BABA pretreatment at each sampling point (*P* < 0.05) (**Figure 1C**). Trypan blue staining also revealed stronger blue staining around the infection sites in inoculated fruit, indicating more extensive pathogen colonization and tissue damage, while BABA+INO samples showed weaker and more localized staining, particularly during the early and middle infection stages (**Figure 1D**). Collectively, these findings suggest that BABA pretreatment effectively delays green mold progression and restricts *P. digitatum* colonization in postharvest lemon fruit.

### 3.2 Transcriptomic analysis reveals BABA-associated reprogramming of phenylpropanoid metabolism in pathogen-infected lemon fruit

To further explore the transcriptional changes associated with BABA-mediated resistance, RNA-seq analysis was performed on peel tissues collected around the wounded or inoculated sites from Control, BABA, Inoculation and BABA+INO fruit at 6 dpi. Principal component analysis showed clear separation among the four treatments, with biological replicates clustering closely within each group (**Figure 2A**). Inoculation separated samples along PC1 (70.63%), whereas BABA+INO and Inoculation were separated along PC2 (8.11%), indicating that BABA modified a defined component of the infection response rather than the overall transcriptional state. Differential expression analysis showed that pathogen inoculation caused broad transcriptional changes, with 3,886 DEGs in INO vs Control and 3,474 DEGs in INO vs BABA (**Figures 2B, C**; **Supplementary Figure S1**). By contrast, BABA alone produced a more limited response, with 531 DEGs in BABA vs Control. The BABA+INO vs INO comparison identified 1,054 DEGs (**Figures 2B, C**).

**Figure 2:**
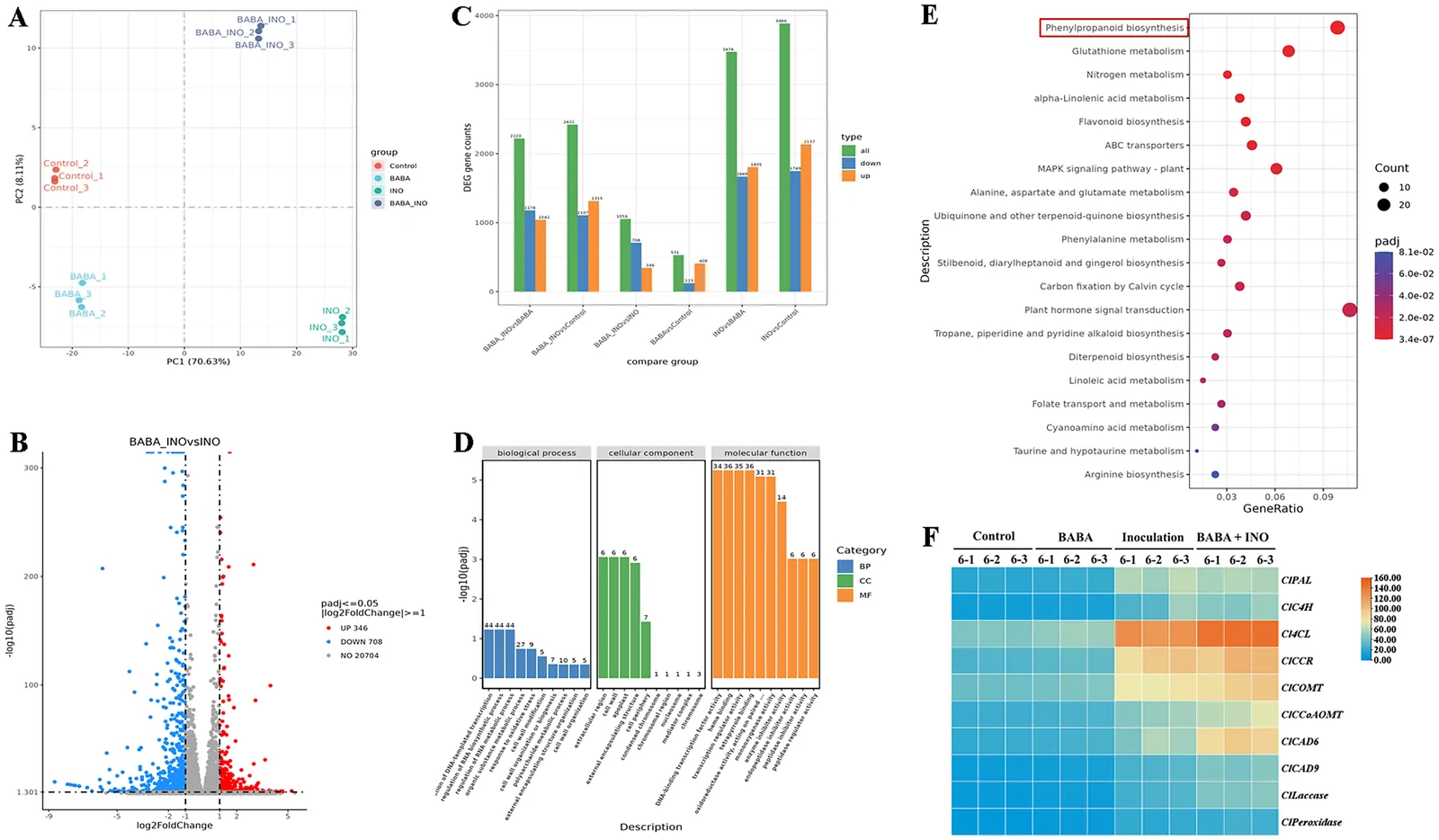
Transcriptome profiling reveals BABA-associated changes in pathogen-responsive gene expression in lemon peel tissues. **(A)** Principal component analysis (PCA) of RNA-seq samples from Control, BABA, INO and BABA+INO treatments. **(B)** Volcano plot showing DEGs between BABA+INO and INO samples. Red and blue dots indicate up- and down-regulated genes, respectively, and grey dots indicate genes without significant differential expression. **(C)** Numbers of DEGs in each pairwise comparison among the four treatment groups. **(D)** GO enrichment analysis of DEGs between BABA+INO and INO samples. The enriched terms are grouped into biological process (BP), cellular component (CC) and molecular function (MF) categories. **(E)** KEGG pathway enrichment analysis of DEGs between BABA+INO and INO samples. **(F)** Heatmap of TPM values for lignin biosynthesis-related genes in the four treatments at 6 dpi (n = 3 biological replicates per treatment).

GO enrichment analysis of the DEGs between BABA+INO and Inoculation showed that these genes were mainly associated with cellular metabolism, catalytic activity and stress-related responses (**Figure 2D**; **Supplementary Figure S2**). KEGG enrichment analysis further indicated that several defense-related pathways were enriched, with phenylpropanoid biosynthesis being particularly prominent among these DEGs (**Figure 2E**; **Supplementary Figure S3**). Consistent with the KEGG enrichment of phenylpropanoid biosynthesis, lignin biosynthesis-related genes displayed a clear treatment-dependent expression pattern (**Figure 2F**). *ClPAL*, *ClC4H*, *Cl4CL*, *ClCCR*, *ClCOMT*, *ClCCoAOMT*, *ClCAD6*, *ClCAD9*, ClLAC (Laccase) and ClPOD (Peroxidase) remained at relatively low levels in Control and BABA-treated fruit, but were induced after pathogen inoculation. Most of these genes showed stronger expression in BABA+INO fruit than in the Inoculation group at 6 dpi. These results indicate that BABA pretreatment strengthens pathogen-induced lignin metabolism, a response that may contribute to cell wall reinforcement and improved resistance in postharvest lemon fruit.

### 3.3 BABA pretreatment modulates the expression of lignin biosynthesis-related genes in infected lemon fruit

To validate the transcriptomic results, ten lignin biosynthesis-related genes were analyzed by qRT-PCR, including *ClPAL* (phenylalanine ammonia-lyase), *ClC4H* (cinnamate 4-hydroxylase), *Cl4CL* (4-coumarate:CoA ligase), *ClCCR* (cinnamoyl-CoA reductase), *ClCOMT* (caffeic acid O-methyltransferase), *ClCCoAOMT* (caffeoyl-CoA O-methyltransferase), *ClCAD6/9* (cinnamyl alcohol dehydrogenases), *ClLaccase* and *ClPeroxidase* (**Figure 3**). Most of these genes remained at relatively low levels in Control and BABA-treated fruit but were induced by *P. digitatum* inoculation. BABA pretreatment further enhanced or prolonged the pathogen-induced expression of several lignin biosynthesis-related genes. *ClPAL*, *ClC4H* and *Cl4CL* showed distinct temporal responses, with the BABA-associated enhancement being more evident at earlier or intermediate stages and diminishing at later sampling points. Among the analyzed genes, *Cl4CL*, *ClCCR*, *ClCOMT*, *ClCCoAOMT*, *ClCAD6* and *ClCAD9* were consistently up-regulated by BABA pretreatment during infection, and their promoters each contained a canonical SMRE motif (ACC[A/T]A[A/C][C/T]) within 2 kb upstream of the start codon (Data S1). These six genes were therefore selected as candidate targets for the subsequent promoter-binding and transcriptional activity assays.

**Figure 3:**
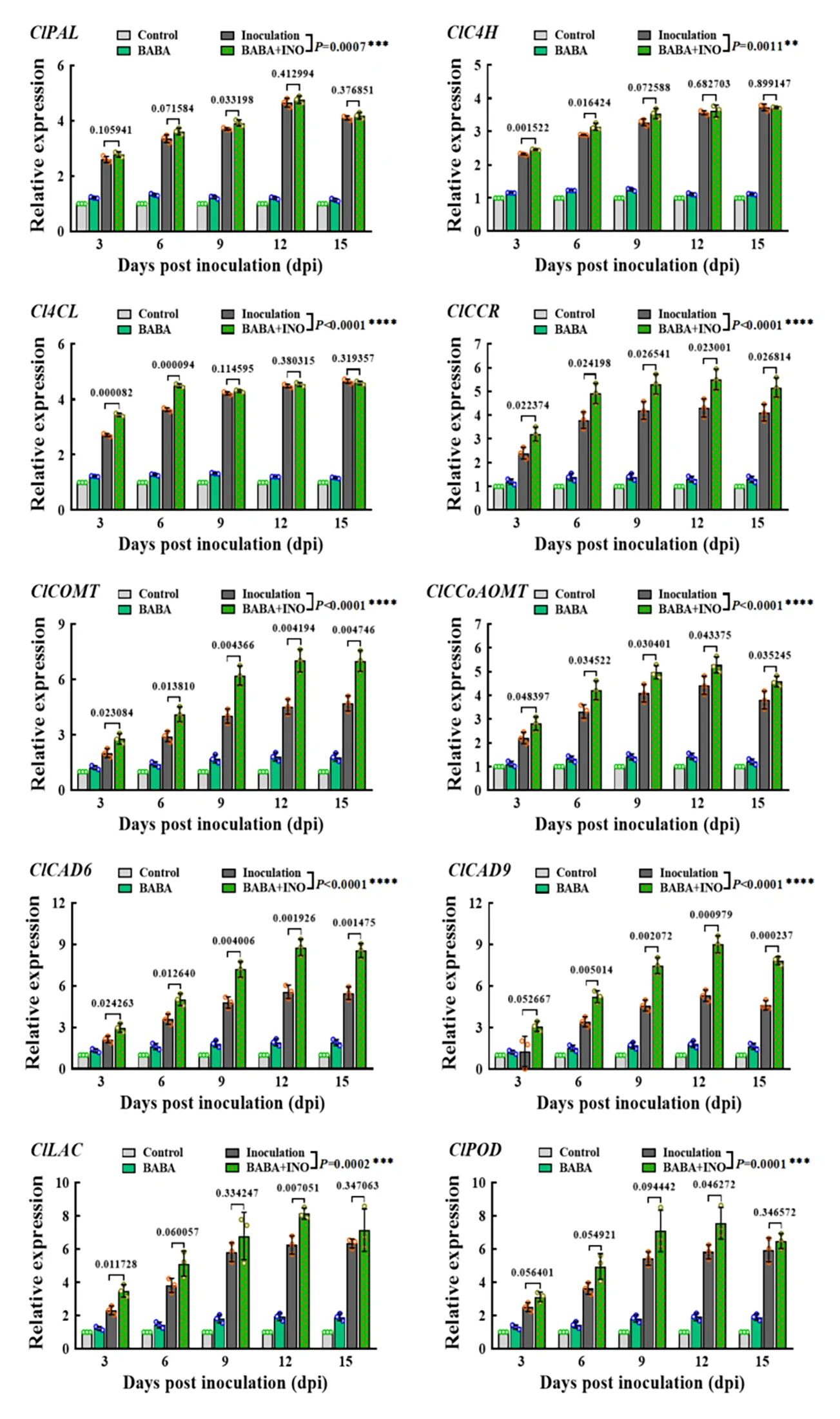
BABA modulates the infection-induced transcription of lignin biosynthesis-related genes in lemon peel tissues. Relative expression levels of *ClPAL*, *ClC4H*, *Cl4CL*, *ClCCR*, *ClCOMT*, *ClCCoAOMT*, *ClCAD6*, *ClCAD9*, *ClLaccase* and *ClPeroxidase* in lemon fruit subjected to Control, BABA, INO and BABA+INO treatments during storage. Peel tissues around the treated sites were sampled at 3, 6, 9, 12 and 15 dpi. Expression levels were determined by RT-qPCR and normalized to the corresponding control value. Data are presented as means ± SD of three biological replicates. A two-way ANOVA was used to test the effects of treatment, time and their interaction. Asterisks and *P* values indicate the significance of the treatment effect (column factor): *P* < 0.05 (*), *P* < 0.01 (**), *P* < 0.001 (***), and *P* < 0.0001 (****). *P* values above the horizontal brackets indicate pairwise comparisons between the Inoculation and BABA+INO groups at individual sampling points, whereas the P values and asterisks beside the bracket in the upper right indicate the overall column factor effect.

The expression of several wound-healing/suberin-associated genes was also analyzed, including *ClCYP86B1* encoding cytochrome P450 family 86B1, *ClGPAT5* encoding glycerol-3-phosphate acyltransferase 5, *ClFAR3*, *ClFAR4* and *ClFAR5* encoding fatty acyl reductases, and *ClFeruloyl transferase* encoding feruloyl transferase (**Supplementary Figure 4**). These genes were induced by pathogen inoculation and generally showed higher expression in BABA+INO fruit than in the Inoculation fruit at several sampling points. Collectively, these findings indicate that BABA pretreatment promotes lignin biosynthesis and may also reinforce wound-related cell wall repair responses in infected lemon fruit.

### 3.4 BABA pretreatment promotes lignin accumulation in infected lemon fruit

To determine whether the transcriptional activation of lignin biosynthesis-related genes was accompanied by lignin accumulation, lignin content and histochemical staining were further analyzed. Lignin content remained relatively low and stable in Control and BABA-treated fruit during storage, whereas *P. digitatum* inoculation markedly increased lignin accumulation (**Figure 4A**). The absence of a difference between Control and BABA-treated fruit indicates that BABA alone did not induce lignin accumulation in the absence of pathogen challenge. BABA+INO fruit showed consistently higher lignin content than the Inoculation group from 6 to 15 dpi (*P* < 0.05), with the highest level observed at 12 dpi (*P* = 0.00427), whereas the two inoculated groups did not differ significantly at 3 dpi.

**Figure 4:**
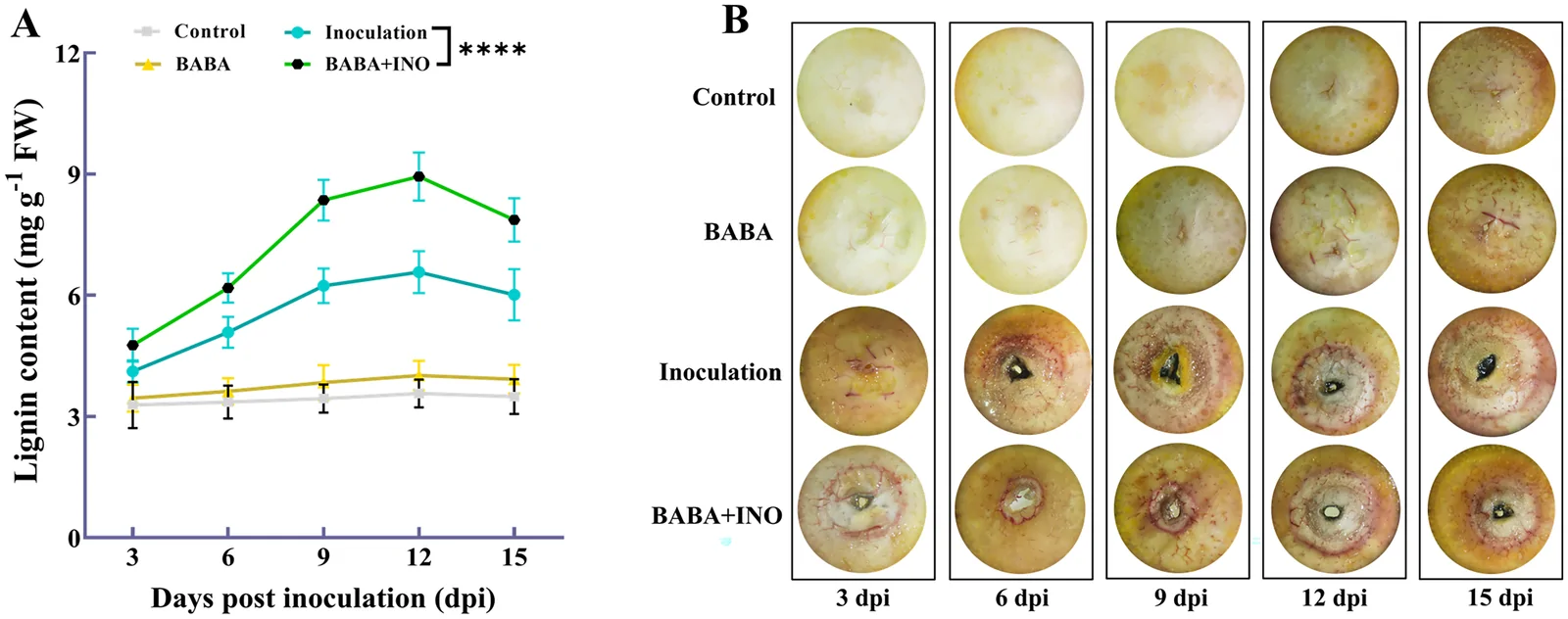
BABA promotes lignin accumulation in lemon peel tissues after *Penicillium digitatum* inoculation. **(A)** Lignin content in lemon peel tissues from Control, BABA, INO and BABA+INO treatments during storage. Samples were collected around the treated sites at 3, 6, 9, 12 and 15 dpi. Data are presented as means ± SD of three biological replicates. Asterisks indicate a significant treatment effect based on two-way ANOVA: ****P < 0.0001. **(B)** Phloroglucinol-HCl staining of lemon peel tissues showing lignin deposition around the treated or inoculated sites. Reddish staining indicates lignified regions.

Histochemical staining further supported these results. Only weak staining was observed in Control and BABA-treated fruit, in which the discoloration at later time points was largely brownish rather than the magenta color characteristic of phloroglucinol-stained lignin (**Figure 4B**). Compared with the Inoculation group, BABA+INO fruit exhibited stronger and more clearly distributed staining around the inoculation area, especially during the middle and late stages of infection. These results indicate that BABA pretreatment enhances pathogen-induced lignin deposition in lemon fruit.

### 3.5 ClMYB46L is a conserved lignin-related R2R3-MYB transcription factor responsive to BABA and pathogen infection

To search for MYB transcription factors potentially involved in lignin regulation, lemon MYB candidates were analyzed together with *Arabidopsis* MYB proteins known to participate in lignin biosynthesis. In the phylogenetic tree, evm_TU_ctg99_12 was placed in the lignin-related R2R3-MYB group and was positioned close to the MYB46/83-type regulators from *Arabidopsis* (**Supplementary Figure 5A**). Protein domain analysis showed that evm_TU_ctg99_12 possessed two conserved SANT/MYB DNA-binding domains, a typical feature of R2R3-MYB transcription factors (**Supplementary Figure 5B**). AtMYB46, AtMYB83, AtMYB58 and AtMYB63 were selected for domain comparison with evm_TU_ctg99_12 according to the phylogenetic clustering. Sequence alignment of the two SANT/MYB domains among evm_TU_ctg99_12 and these four *Arabidopsis* lignin-related MYB proteins showed that both domains were highly conserved, with overall consistency values of 76.86% for SANT1 and 80.00% for SANT2 (**Supplementary Figure 5C**). Among the four *Arabidopsis* MYBs, evm_TU_ctg99_12 showed high similarity to the MYB46/83-type regulators, with the closest relationship to AtMYB46 in the phylogenetic analysis. Accordingly, evm_TU_ctg99_12 was designated ClMYB46L.

qRT-PCR analysis showed that *ClMYB46L* maintained low expression levels in Control and BABA-treated fruit, whereas its expression was clearly induced by *P. digitatum* infection (**Supplementary Figure 5D**). Moreover, BABA-pretreated infected fruit showed higher *ClMYB46L* transcript abundance than the Inoculation group throughout the storage period, with a highly significant main effect of treatment (*P* < 0.0001). These results indicate that *ClMYB46L* is a conserved lignin-related R2R3-MYB transcription factor and may be associated with BABA-enhanced lignin-mediated defense in infected lemon fruit.

### 3.6 ClMYB46L directly activates lignin biosynthesis-related genes

Based on the expression patterns shown in **Figure 3**, *Cl4CL*, *ClCCR*, *ClCOMT*, *ClCCoAOMT*, *ClCAD6* and *ClCAD9* were selected as representative BABA-responsive lignin biosynthesis-related genes. Their 2,000 bp upstream regions contained putative SMRE-like MYB-responsive elements, which were used for EMSA and Y1H assays (Supplementary Data 1 and 2). EMSA assays showed that recombinant ClMYB46L bound to the biotin-labeled promoter fragments of *Cl4CL*, *ClCCR*, *ClCCoAOMT*, *ClCOMT*, *ClCAD6* and *ClCAD9*, as indicated by shifted DNA–protein bands. The shifted bands were markedly reduced by excess unlabeled wild-type competitor probes, whereas unlabeled mutant competitors failed to compete effectively. These results support the specific recognition of the predicted SMRE-like motifs by ClMYB46L (**Figure 5A**).

**Figure 5:**
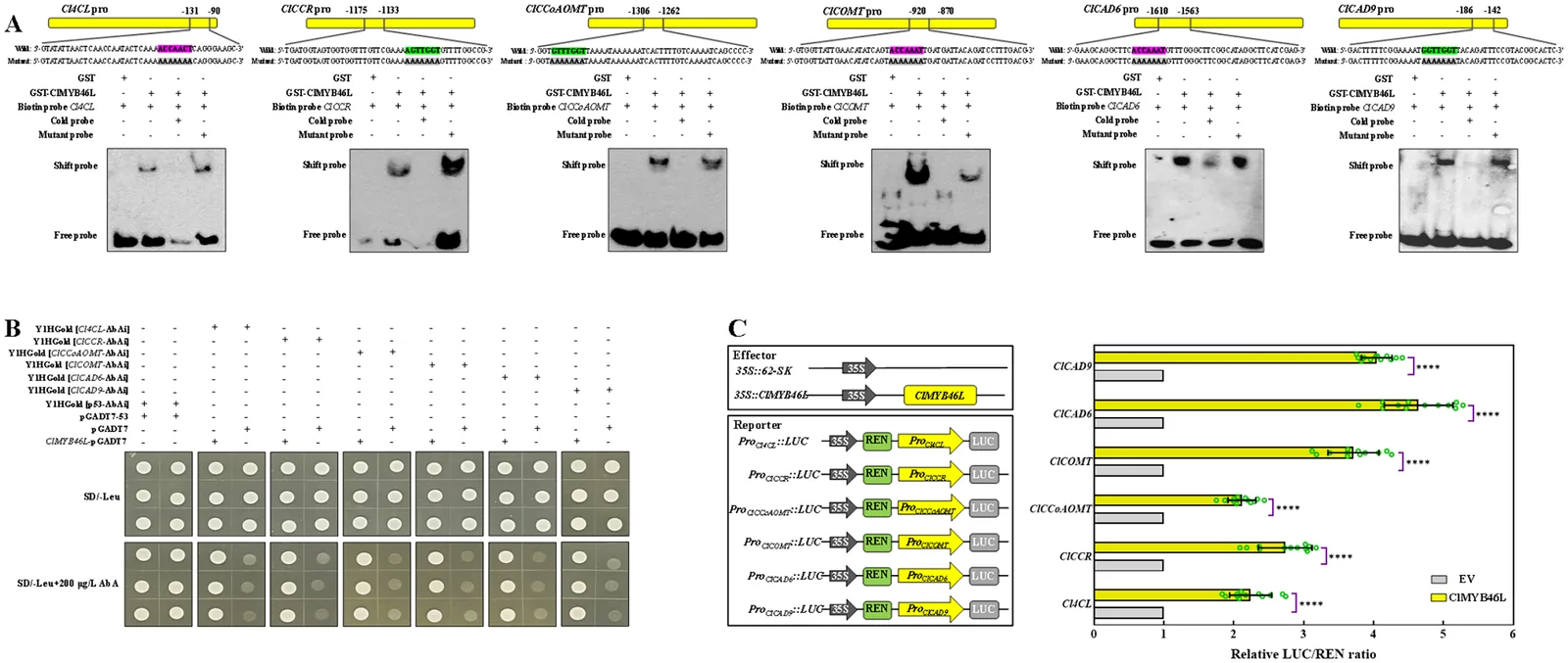
ClMYB46L binds to and activates promoters of lignin biosynthesis-related genes. **(A)** EMSA analysis of the interaction between ClMYB46L and promoter fragments of *Cl4CL*, *ClCCR*, *ClCCoAOMT*, *ClCOMT*, *ClCAD6* and *ClCAD9*. Biotin-labeled probes containing the predicted MYB-binding sites were used for binding assays. Unlabeled probes were used as competitors, and mutated probes were used to test binding specificity. **(B)** Yeast one-hybrid analysis of ClMYB46L binding to the corresponding promoter elements. Yeast growth on SD/-Leu medium supplemented with AbA indicates protein–DNA interaction. The p53-AbAi/pGADT7–53 combination served as the positive control. **(C)** DLR assay showing the activation of lignin biosynthesis-related gene promoters by ClMYB46L in *Nicotiana benthamiana* leaves. The reporter and effector constructs are shown on the left, and relative promoter activity is expressed as the LUC/REN ratio on the right. Data are presented as means ± SD. Asterisks indicate significant differences compared with the empty vector control: ****P < 0.0001.

Yeast one-hybrid assays further confirmed the interaction between ClMYB46L and the promoters of these six genes. Yeast cells co-transformed with ClMYB46L and the corresponding promoter fragments grew on selective medium containing AbA, whereas the negative controls showed no obvious growth, indicating that ClMYB46L could bind to these promoter regions in yeast cells (**Figure 5B**).

Dual-luciferase reporter assays were then used to examine the transcriptional activation of these promoters by ClMYB46L. Relative to the empty-vector control, ClMYB46L significantly increased promoter-driven LUC/REN ratios for Cl4CL, ClCCR, ClCCoAOMT, ClCOMT, ClCAD6 and ClCAD9 (**Figure 5C**). These results indicate that ClMYB46L directly binds to and activates multiple lignin biosynthesis-related genes.

### 3.7 Transient overexpression of ClMYB46L enhances lignin-associated resistance in lemon fruit

Transient overexpression assays were performed to evaluate the function of *ClMYB46L* in lemon fruit. After *P. digitatum* inoculation, empty-vector control fruit developed obvious necrotic lesions, whereas *ClMYB46L*-overexpressing fruit showed smaller infection areas across four independent overexpression batches (**Figure 6A**). qRT-PCR confirmed that *ClMYB46L* transcript levels were markedly increased in OE-1 to OE-4 compared with EV, indicating successful transient overexpression (**Figure 6B**). Consistently, lesion diameter was significantly reduced in *ClMYB46L*-overexpressing fruit relative to EV (**Figure 6C**). Trypan blue staining further showed weaker blue staining in OE fruit, suggesting reduced pathogen colonization and tissue damage (**Figure 6D**). This was also supported by the fungal biomass assay, in which all four OE batches showed significantly lower relative fungal biomass than EV (**Figure 6E**).

**Figure 6:**
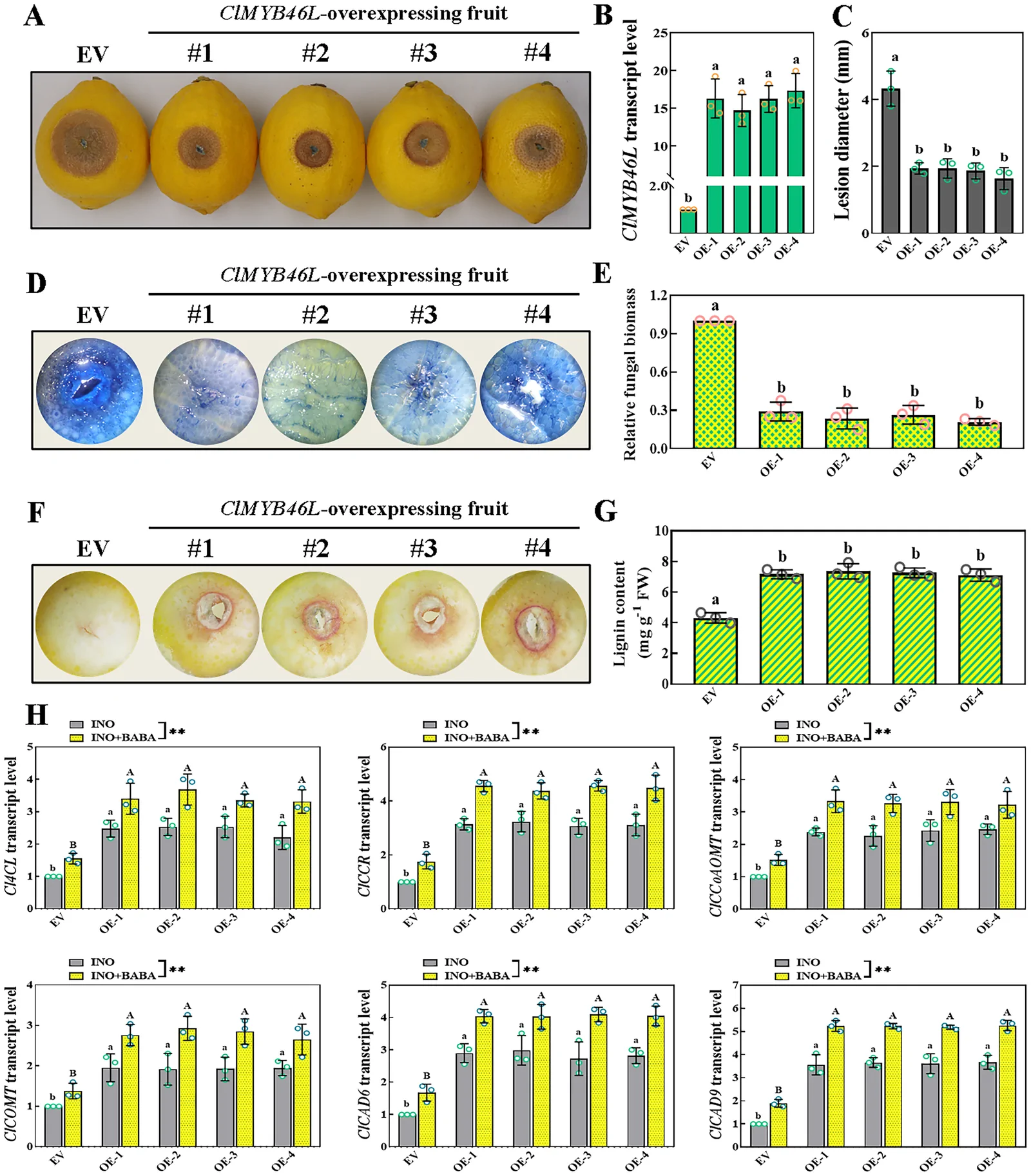
Transient overexpression of ClMYB46L enhances lignin-associated resistance in lemon fruit. **(A)** Representative lesion phenotypes of empty vector (EV) and ClMYB46L-overexpressing lemon fruit after *P. digitatum* inoculation. Four independent overexpression batches are shown as OE-1 to OE-4. **(B)** Relative transcript levels of ClMYB46L in EV and ClMYB46L-overexpressing fruit. **(C)** Lesion diameter in EV and ClMYB46L-overexpressing fruit after pathogen inoculation. **(D)** Trypan blue staining of peel tissues showing infection-associated staining around the inoculation sites. **(E)** Relative fungal biomass in EV and ClMYB46L-overexpressing fruit. **(F)** Phloroglucinol-HCl staining showing lignin deposition around the inoculation sites. **(G)** Lignin content in EV and ClMYB46L-overexpressing fruit. **(H)** Relative expression levels of lignin biosynthesis-related genes in EV and ClMYB46L-overexpressing fruit under INO and INO+BABA conditions. Data are presented as means ± SD of three biological replicates. In **(B, C, E, G)**, different letters indicate significant differences among EV and OE fruit (*P* < 0.05). In **(H)**, lowercase and uppercase letters indicate significant differences within the INO and INO+BABA conditions, respectively.

Histochemical staining showed stronger lignin-associated signals around the inoculated wounds of *ClMYB46L*-overexpressing fruit than in EV (**Figure 6F**). Lignin quantification further showed that OE-1 to OE-4 accumulated significantly more lignin than EV (**Figure 6G**). In addition, *ClMYB46L* overexpression increased the transcript abundance of lignin pathway genes, including *Cl4CL*, *ClCCR*, *ClCCoAOMT*, *ClCOMT*, *ClCAD6* and *ClCAD9*, under pathogen-inoculated conditions. Compared with INO alone, INO+BABA generally further enhanced these gene transcripts, with higher levels detected in *ClMYB46L*-overexpressing fruit than in EV (**Figure 6H**). These findings indicate that *ClMYB46L* promotes lignin pathway gene expression and lignin accumulation, thereby strengthening lemon fruit resistance to *P. digitatum*.

## 4 Discussion

BABA has been generally regarded as a defense-priming compound that strengthens host resistance by improving the capacity of plant tissues to respond to subsequent pathogen challenge (Conrath et al., 2006; Cohen et al., 2016; Thevenet et al., 2017). In the present study, BABA pretreatment restricted green mold progression and limited *P. digitatum* colonization in inoculated lemon peel tissues (**Figure 1**). These observations suggest that BABA-mediated protection was not simply reflected by delayed symptom appearance, but was also associated with reduced pathogen accumulation and less tissue damage around the infection site. This is consistent with an earlier report on the same pathosystem (Li et al., 2026a), confirming that BABA is effective against green mold in postharvest lemon. Similar BABA-induced protection has been reported in other fruit–pathogen systems. For example, BABA treatment conferred long-lasting postharvest resistance against *Botrytis cinerea* in tomato fruit (Wilkinson et al., 2018), and reduced anthracnose development caused by *Colletotrichum gloeosporioides* in mango fruit (Zhang et al., 2013). Together with these previous findings, our results further support the protective role of BABA in harvested fruit tissues during fungal infection.

At the transcriptional level, BABA pretreatment did not appear to cause a general amplification of the infection response. Instead, the clear separation between BABA+INO and INO samples, together with the DEG enrichment pattern, suggests that BABA selectively modified part of the pathogen-responsive program in infected lemon peel. Among the enriched pathways, phenylpropanoid biosynthesis was particularly prominent, and several lignin biosynthesis-related genes showed stronger induction in BABA-pretreated fruit after *P. digitatum* inoculation (**Figure 2**). Similar links between induced resistance and phenylpropanoid activation have also been observed in other harvested fruit. For example, hot-air treatment enhances the resistance of cherry tomato fruit by activating the phenylpropanoid pathway (Wei et al., 2017), whereas H_2_S fumigation reduces the susceptibility of postharvest navel orange fruit to *Penicillium italicum* through stimulating phenylpropanoid metabolism (Huang et al., 2023). In our study, this transcriptional shift was accompanied by increased lignin accumulation and stronger phloroglucinol-HCl staining around inoculation sites (**Figures 3** and **4**). These results suggest that BABA-associated transcriptional reprogramming was closely linked to localized lignin deposition around the infection site, providing a structural basis for the enhanced resistance observed in BABA-pretreated fruit.

Such localized lignin deposition is consistent with the wound-dependent infection pattern of *P. digitatum* on citrus fruit. As a typical wound-invading pathogen, *P. digitatum* usually enters through damaged peel tissues and then expands from these sites by promoting host tissue maceration, a process closely associated with fungal pectinase activity (Vilanova et al., 2018). Lignification around the inoculation site may therefore strengthen the tissues where fungal colonization begins and slow early lesion expansion. This effect may be associated with the reduced susceptibility of lignified cell walls to enzymatic degradation and pathogen penetration (Bhuiyan et al., 2009; Liu et al., 2018), which could help explain the lower fungal biomass and slower disease development observed in BABA-pretreated fruit. The coordinated induction of suberin biosynthetic genes in BABA-pretreated infected fruit indicates activation of the wound suberization program (**Supplementary Figure 4**). In postharvest citrus, this program has been shown to reduce decay by promoting suberin deposition and cell wall reinforcement around damaged tissues (Gao et al., 2024), suggesting that BABA strengthens barrier formation at the infection site. These lignin- and wound repair-related changes indicate that BABA may enhance local barrier formation in wounded peel tissues, thereby limiting *P. digitatum* invasion at the inoculation site. Notably, these responses were weak in BABA-treated fruit without pathogen inoculation, but became much stronger when BABA pretreatment was followed by pathogen challenge. This infection-dependent pattern supports a priming-like mode of action, in which BABA prepares wounded tissues to activate a stronger structural response once infection occurs, rather than continuously activating defense before pathogen attack.

R2R3-MYB transcription factors have well-established roles in lignin biosynthesis and secondary wall formation (Tian et al., 2013). *Arabidopsis* MYB46 and MYB83 are representative upstream regulators of secondary wall development and have been linked to the activation of lignin biosynthetic genes (Wang and Dixon, 2012). In this study, ClMYB46L clustered with the *Arabidopsis* MYB46/83-type regulators and contained two conserved SANT/MYB domains, supporting its annotation as a lignin-related R2R3-MYB transcription factor (**Supplementary Figures 5A–C**). Notably, ClMYB46L showed little response to BABA alone, whereas its transcript abundance increased after *P. digitatum* challenge and reached higher levels in BABA+INO fruit (**Supplementary Figure 5D**). This expression pattern paralleled lignin accumulation in infected peel tissues, suggesting that ClMYB46L may participate in BABA-enhanced lignification. Similar roles of MYB factors in defense-associated lignification have been reported in other plant systems. In *Arabidopsis*, MYB15 contributes to pathogen-induced lignin deposition during effector-triggered immunity (Kim et al., 2020), whereas CmMYB15 improves aphid resistance in chrysanthemum through the regulation of lignin pathway genes (An et al., 2019). These studies suggest that MYB-mediated lignin regulation is not only related to developmental secondary wall formation, but can also be recruited for inducible defense responses. In lemon fruit, the infection-responsive induction of ClMYB46L provides a regulatory link between BABA-enhanced transcriptional changes and lignin-associated cell wall reinforcement.

Promoter-binding and transient overexpression assays further support ClMYB46L as a positive regulator of lignin-associated resistance. ClMYB46L bound to and activated the promoters of several lignin biosynthetic genes, including *Cl4CL*, *ClCCR*, *ClCCoAOMT*, *ClCOMT*, *ClCAD6* and *ClCAD9* (**Figure 5**). These genes participate in different steps of monolignol biosynthesis, from hydroxycinnamoyl-CoA formation to methylation and reduction reactions leading to lignin precursor production (Boerjan et al., 2003; Vanholme et al., 2010). Therefore, ClMYB46L appears to act at a pathway level by coordinating a set of lignin biosynthetic genes rather than regulating a single downstream reaction. Similar pathway-level regulation has been reported for EjMYB8 in loquat, where this MYB factor activates lignin biosynthetic genes during flesh lignification (Wang et al., 2016), and for the EjERF39–EjMYB8 module, which enhances *Ej4CL1* activation during cold-induced lignification (Zhang et al., 2020b). In the present study, transient overexpression of ClMYB46L reduced lesion development and fungal biomass while increasing lignin accumulation and lignin-related gene expression in lemon fruit (**Figure 6**). These results support a link between ClMYB46L-mediated transcriptional activation, lignin accumulation and resistance in harvested lemon tissue. The stronger lignin-related response under BABA+INO conditions further suggests that BABA may reinforce this ClMYB46L-associated lignification response during infection, although the link between BABA treatment and ClMYB46L induction remains to be clarified. Thus, ClMYB46L represents an important transcriptional component linking BABA-associated transcriptional reprogramming with lignin biosynthesis and cell wall reinforcement.

## 5 Conclusion

Taken together, this work supports a model in which BABA primes wounded lemon peel for a stronger lignin-associated barrier response after *P. digitatum* infection (**Figure 7**). In this model, BABA alone causes only limited structural activation, but enables infected tissues to mount a stronger phenylpropanoid/lignin response together with wound-healing/suberin-associated changes around the inoculation site. ClMYB46L acts as an important transcriptional component of this response by activating multiple lignin biosynthetic genes and promoting lignin accumulation, thereby contributing to local cell wall reinforcement and disease restriction. This mechanism is well suited to a wound-invading pathogen such as *P. digitatum*, whose colonization begins at damaged peel tissues and depends on rapid lesion expansion. How BABA treatment promotes ClMYB46L induction remains unclear, and future work should determine how BABA perception, wound signals and pathogen challenge converge to regulate ClMYB46L-mediated lignification in harvested citrus fruit.

**Figure 7:**
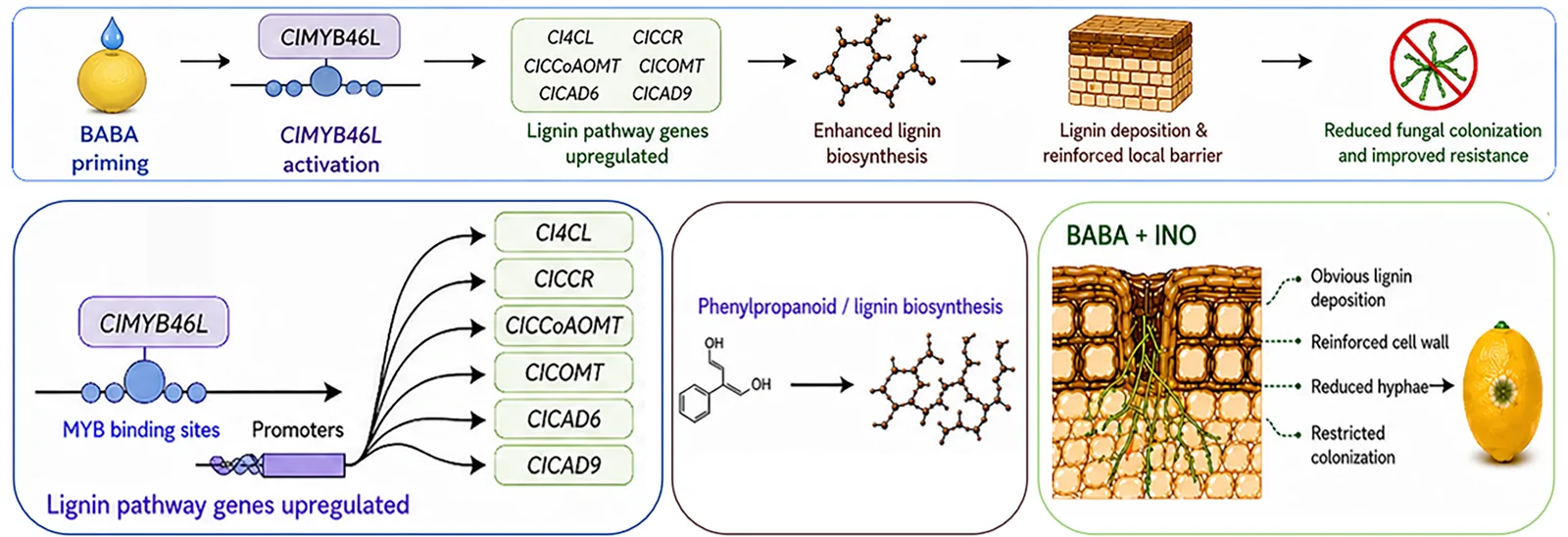
BABA primes lignin-associated resistance in postharvest lemon fruit. BABA pretreatment before *P. digitatum* inoculation reshapes pathogen-responsive transcription and promotes phenylpropanoid/lignin biosynthesis in lemon peel. ClMYB46L activates multiple lignin biosynthetic genes, resulting in enhanced lignin accumulation, reinforced local cell walls, restricted fungal colonization and improved disease resistance.

## Data Availability

The datasets presented in this study can be found in online repositories. The names of the repository/repositories and accession number(s) can be found below: https://www.ncbi.nlm.nih.gov/,PRJNA1520993.

## Supplementary material

Supplementary Figure 1 Volcano plots showing DEGs among different treatment comparisons. **(A)** BABA+INO vs BABA. **(B)** BABA+INO vs Control. **(C)** BABA vs Control. **(D)** INO vs BABA. **(E)** INO vs Control. Red dots indicate up-regulated genes, blue dots indicate down-regulated genes, and grey dots indicate genes without significant differential expression. Differential expression was defined using an adjusted *P* < 0.05 and |log_2_(fold change)| ≥ 1.

Supplementary Figure 2 GO enrichment analysis of DEGs from different treatment comparisons. **(A)** BABA+INO vs BABA. **(B)** BABA+INO vs Control. **(C)** BABA vs Control. **(D)** INO vs BABA. **(E)** INO vs Control. Enriched GO terms were classified into biological process (BP), cellular component (CC) and molecular function (MF) categories. The y-axis indicates −log_10_(adjusted *P* value), and the numbers above the bars indicate the number of genes assigned to each term.

Supplementary Figure 3 KEGG pathway enrichment analysis of differentially expressed genes from different treatment comparisons. **(A)** BABA+INO vs BABA. **(B)** BABA+INO vs Control. **(C)** BABA vs Control. **(D)** INO vs BABA. **(E)** INO vs Control. The bubble size represents the number of genes enriched in each pathway, and the color scale indicates the adjusted *P* value. The x-axis shows the gene ratio for each enriched pathway.

Supplementary Figure 4 Expression profiles of wound-healing and suberin-associated genes in lemon peel tissues under different treatments. Relative transcript levels of *ClCYP86B1*, *ClGPAT5*, *ClFAR3*, *ClFAR4*, *ClFAR5* and *ClFeruloyl transferase* were analyzed in Control, BABA, INO and BABA+INO fruit at 3, 6, 9, 12 and 15 dpi. Expression values were determined by RT-qPCR and normalized to the corresponding control value. Data are presented as means ± SD of three biological replicates. Asterisks and *P* values indicate the significance of the treatment effect from two-way ANOVA: *P* < 0.0001 (****).

Supplementary Figure 5 Identification and expression analysis of the lemon MYB46-like transcription factor ClMYB46L. **(A)** Phylogenetic analysis of lemon MYB candidates and *Arabidopsis* lignin-related MYB proteins. The lemon gene evm_TU_ctg99_12 clustered close to AtMYB46/83-type regulators and was designated *ClMYB46L*. **(B)** Conserved domain organization of ClMYB46L and representative *Arabidopsis* lignin-related MYB proteins. SANT/MYB domains are indicated in red. **(C)** Multiple sequence alignment of the two SANT/MYB domains among ClMYB46L and *Arabidopsis* MYB proteins. Conserved residues are highlighted, and the consistency values are shown above the alignments. **(D)** Relative expression of *ClMYB46L* in Control, BABA, INO and BABA+INO fruit at 3, 6, 9, 12 and 15 dpi. Expression values were determined by RT-qPCR and normalized to the corresponding control value. Data are presented as means ± SD of three biological replicates. Asterisks and *P* values indicate the significance of the treatment effect from two-way ANOVA: *P* < 0.0001 (****).

Supplementary Table 1 Oligonucleotide primers used for molecular analyses in this study.

Supplementary Data Sheet 1 Promoter sequences of lignin biosynthesis-related genes and predicted MYB-responsive elements. The 2,000 bp upstream promoter sequences of *Cl4CL*, *ClCCR*, *ClCCoAOMT*, *ClCOMT*, *ClCAD6* and *ClCAD9* are shown. Putative MYB-binding sites were predicted according to the secondary wall MYB-responsive element (SMRE) consensus motif, ACC(A/T)A(A/C)(C/T). SMRE-like motifs identified on the forward strand are shown in red bold, whereas motifs identified on the reverse-complement strand are shown in green bold.

Supplementary Data Sheet 2 Y1H bait sequences containing predicted MYB-responsive elements. Approximately 50 bp promoter fragments surrounding the predicted SMRE-like motifs were selected from *Cl4CL*, *ClCCR*, *ClCCoAOMT*, *ClCOMT*, *ClCAD6* and *ClCAD9*. For Y1H analysis, three tandem copies of each fragment were synthesized as bait sequences. Putative SMRE-like motifs predicted from the forward strand are shown in red bold, whereas those predicted from the reverse-complement strand are shown in green bold. Restriction sites introduced for cloning are underlined.
