# Supplementary material for: β-aminobutyric acid primes lignin-associated resistance to Penicillium digitatum in postharvest lemon fruit involving ClMYB46L-mediated transcriptional activation
Source: Front Plant Sci. 2026 Oct 28;17:1984112. doi: 10.3389/fpls.2026.1984112 (PMC13752077; doi:10.3389/fpls.2026.1984112)

Supplementary material

Supplementary Figure 1

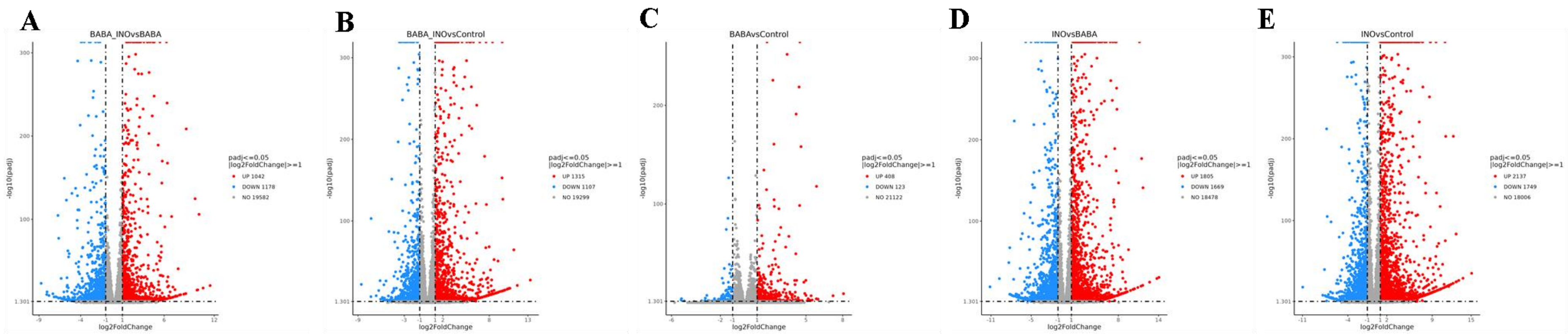

Supplementary Figure 2

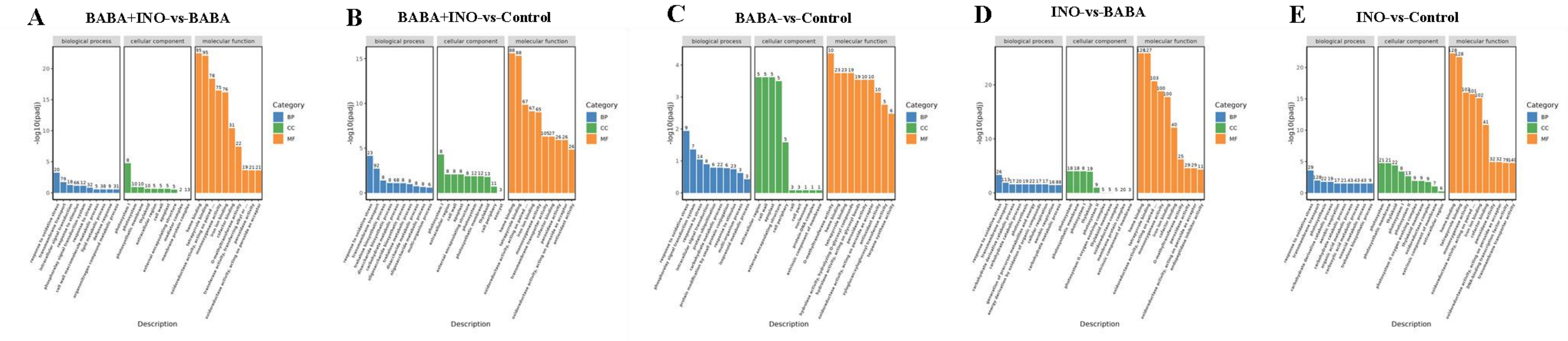

Supplementary Figure 3

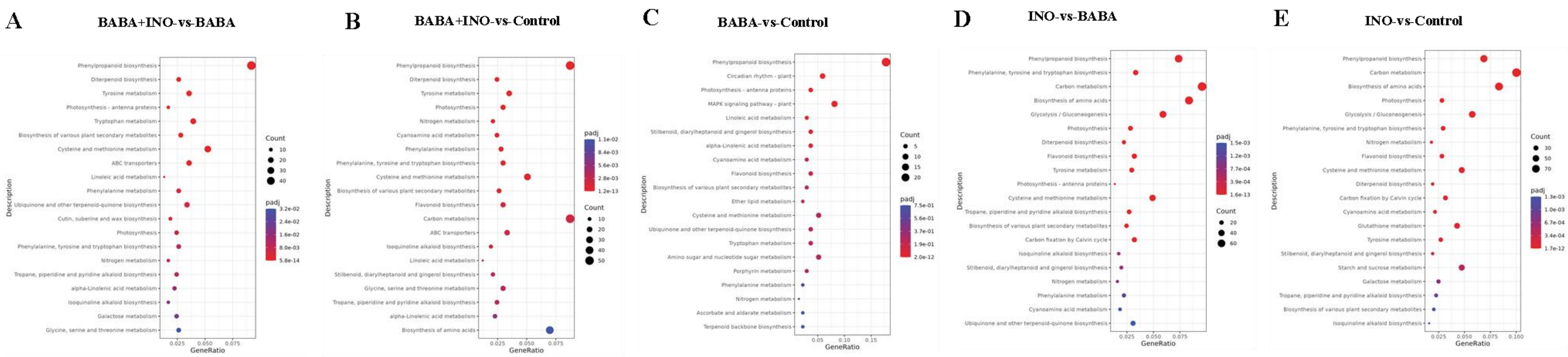

Supplementary Figure 4

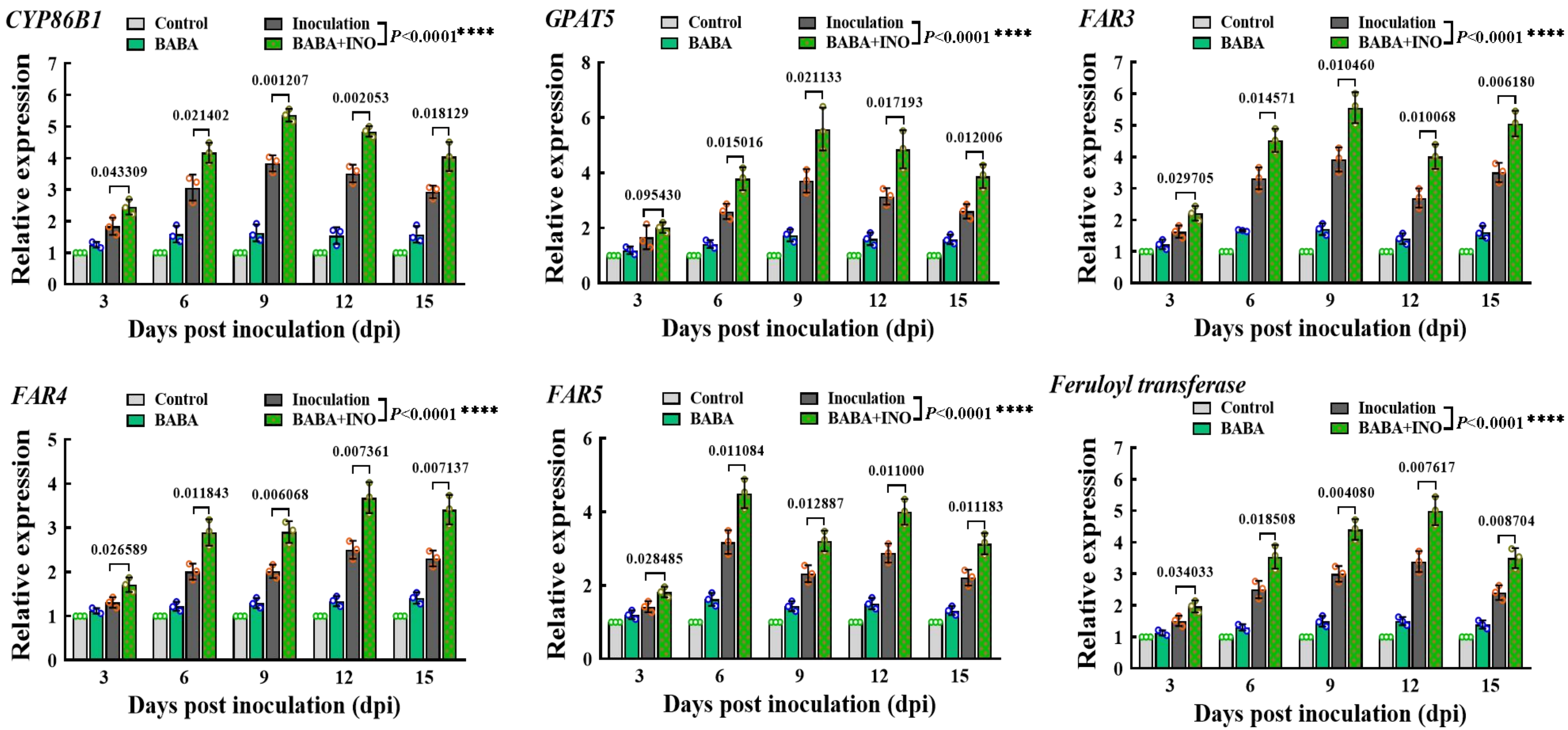

Supplementary Figure 5

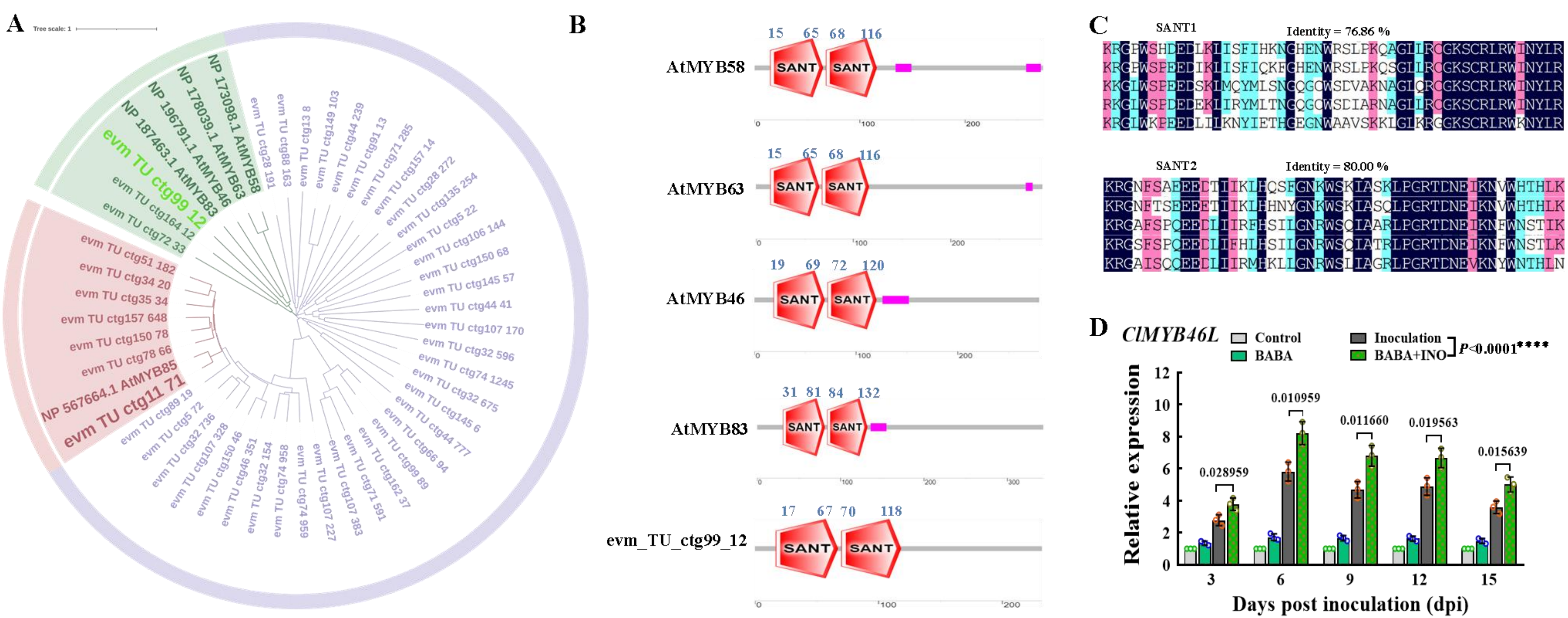

Supplementary Data 1

Cl4CL (evm_TU_ctg86_262) promoter:

GATGATACAACCATAAACTCTTGTATAAACTTATCTTGTACAAACGGATGTGTCATTAATTCATTGGTTGAATGAAAATATAAAATAATAGAAATAAATCA
TGTGGGCCAAGAGATATTTTATTCAACCAATGAATTAACGACACATCAGTTTGTACAAAATAAGTTTGTACAAGAGTTTGTGGCTATATCATTACTCATAA
TAATAATAATAACAAGAAGAAGAAGAAGAAGAAGAAGTTAGGGGCATTGTTTATTCATTAAATTATCCTTAAATTTTAAATTTACGCAAATGTTATTTAAT
TATATTTGAGAGTTTTATTTTAAAATAGTTCGTATGTATTTAATTAAAAATCATGTTTTGCCTTTAATGATTATACACTAAAATAATATTAAATTAACGAACG
AGAGTTTTGTGCTATTATTTAAATAATAATGATGAAGTGGGCTATTACATCAAACCTTAAGGGTGAAAATGATTTTTTTCCCTTTTCAAAACTCTATTATAA
TCTTCCAAAAGTATCTATTAAGCCTGTTTTAGCAATGGCGATTACTTGCCAAATATATGTTTACCAGTCTAGTAAGGATGAAAAATAATTTTTATTATCCAT
TAATAGGCAATAGTTATACTGTGTGTGGATGTGGGTTCATGTTAACAAAAAAAAATTGAGGATACTAAAATATATACATATATATATATATCAATATATTAATG
GATAAGGAAAAAGAAAGCACATCCTGTAATATATGATTTTTTTTTTGTATTTAATGACATCTTTATTTTTTGTAAGATCACAAACTTTATTAAAATTTAAGA
AAATTATTACACCATGTATTGTAATAGTACAATAGCATTACACTACATTATTACAATATATGGTGTAATAGTTTTCTTAAATTTTATTCCAATTTATATATAAA
GTATCATTGGATTCAAATTTGATGATCCTTTATTTATTTCGAAGTAAGGGGGAAAAAAAAGGTTCCAGATCCCGAATTGATTACATATGTGTGTGTGTTTG
TCTGACATTAGTATTACTCTCTCTTGAACTACTTTAGCGGGGAAATTAAAATCCTTTATGACCACAATCAAATTGAGTGGTTGTAGACTGGTGTGTGGTG
TC**GTTTGGT**GCAAAACGACCGCCTCATCACTACCCCAAAGAGGGTTAAAGTACGCACATGCAATGCCCACATAACTGGTAAGTCACATCCTCCGTGGC
TTATGATTGGTGGCTTACAGTCAAAGCTGATGACTTTCACGCACCATAGTCTATCCCCAAGTACGCCCTCTCACGTAACCCCATTTCCAAGAAGAGCAC
GACGGATGGAACGGTTCTTGTAAAATTACATCATAAAATCAATTTCTTTGATGATTGTCAAATGATACTGTAAAATTACTACTGCCTGATTTAATAATATAA
AGATAAATGTTAATCAACAATGTAAGGATATCAGCACCTCCAATGTTTGACTCAACAAATAATTAAACTCTCATGTTTTAAAAATGGACAGTTAAATTTG
ATTCGTTTTAAAAACAGATATTCATACTTTGTAAATTTTATCTATTTAAATGGATATTTAGTGTTAATGTGTAATGTCAATATGATAAGTTAATGCATGCAAT
CCTAGAATGCCACATTCGTGGTACAAAACAAGCAAGAAAATTTTTATTTAACGACAAAAATTTGTTCTTCGTGTAATATGAGAGTATGGGTTGTATTGGA
AATATATATGGTAAGATATATACGTAATATTTAATAAATAGCACCCTATTTTAAGAAAACTGATTGTCAATTTCTTCTCTTGACAAATTCTAAATCAACTACT
CAATCAATCTCACCACCTCCTTTTCTTCTACCATC**ACCTACT**TTGTATATTAACTCAACCAATACTCAAA**ACCAACT**CAGGGAAGCAAAAAAA**ACCAA
AC**ACTCACCATCCCCTTTGCTTCCCAAAATTTCTCTCTCTCTCTCATTTTTCTCATATCCATATCCTCTTTGCAAAA

ClCCR (evm_TU_ctg170_30) promoter:

ACTTTTGTATTAATGTCCATCCAATCACTCCAATGGCGTAAAATAAATTCTTTTATCAACAATTTCTCTAATAATGCCTTCTCGTACTGTTCGCTTCGAATT
TCAACATAGTTGACGAAAAACAATTTCTTCTCATAATCCACTAACTGTTGAAAAATATTACAATTCTTTTTGTCTTCTTTTGGTAAATCAAGAGAATAACA
AGAGATTGTATCAACAACTACCTCTTTTTCCTCGAGAAAATCAAAAGATGTATGAACAATTATCTCTTCTTGTCCCACAATATCAAGATACTCATCAAATT
TCAGTAGTTGATCCAAATCAATATCTTTCTGTTGAGAATTTGTTGTAAACACGAATGGCCAATGAATATTCTTCTCAGAAGGAATAATATTGTCGAGTGA
AACAAAGTTACTATTTTCCTCATTATCCTCATAACAAACATCATCACGAACAACTTCATCAAATCCATATGCTTGCTTCATGTGCAAAGATTTTCTATTTTG
AAGTTTCTCTAAATCTTGAGGAATCGAACGTAAAATCCCCAACATCCTAATTAAAACTTCTTTCAAGATAAAATCTCCACGGTGATGTTGTGGTTTCATG
ATTCAATTTTTTTTTTGTTTTCTCTCTCATTTTTTCCTTCTTTTTTTTTGGTCTTTGTTTGGGTTCTTTTGCTAAATTCAGAAAACAAAAGGGCAATAATGG
TATAAATGAAAGAATTGGGTCAGAATTGTGAATAAGCAAAAGATAGGGTTATTGGCAACGGTGATTAGAAATTTGGACAAGGATGGCCAGATCTAAAA
GATTTTGGGTCGGTGAGTATGATGGTAGTGGTGGTTTGTTCGAAA**AGTTGGT**GTTTTGGCCGAAATTTGGAGATAAAGGCGGCGGTTTTGTTGTTGGT
TTCGATTTCGGCAATCAATTAGTCGATCAAAGGTGGGGAATAATAAAAGAGGTTGAGCAACGTTAGTCTTCACTTTGCTCTGATACCAAGTTGATGCAG
CAAAAATTTATTGTTTAAAATATTTCGTTGATTTTAGAGAATATCGGAATAAAAGAACAACTAAATTCATGTTGATTTAAAAGAATACCGCGAATTTAGGG
GTTAAGACTCATTGATTCCGCAGAATACTGAGAATTAACTGCTCAAATACTCACAAAAAGATTTGAAAATTGAAAATATTATTGGAGTTGAACATGCCTT
CAAAGGCTACAAGAGGGAGCTATTTATATTAGTAGTAATTATCCTAAATCATTAAAGAAAAATAAAATTTTAAATTGGAAAGGAAATTAAAAGTCCTTAA
TTAATTAAAATATATATCCTAGTATAAATTCCTATTCTGCACGAGTAACCAAGATAAATTTTGGGACACCATGTCATTACCCTTTGTAATATTTGAGTGATTT
AAAGAACAATATTATATGTACCAAAAATAACGTAAAACTTAATATAAAATCTTTTTTTTGGTTGCTTTCTGACAATGATCATTGTTATAATTATAATTTTATA
TTAAGTCGATTATGTTGAAATCACTCTAAAATAAAGTATATTTTTATAATTATATAATTAAAATTATAATATATTTTTTATTATTTATTACTTTATACGTATAACG
GTAATTTACACCCTACTTTGGAGTATCTCTAAAATAGCCTTTTTGTTTGACTTCTGCTGCTTTTTAATTTTTTCTTATCCTGTAATTGTTTCATGGCGTAAAT
ATGTTGTGGTCAATATATATATTTATATATTCTTACGCAAAAGTACTCAACGAGTCAGCGTTATTTTTAATGTTACTATTATTAATCTGAGACGAACGACAA
GTCATACACTAGTCACTCAATCTCTCTTAAGTGTTCATCATCGTCATCCAAAATGCAAAATGATCATTACAAAACATTATCCCAATCCATCCAGTTCATCA
TCATCATATTCTCTGACTATAAAATCCCAATCACTGTAATCCCGAACGCTCAAGTCCATTTTTTTTTTCTTTTACAGA

ClCCoAOMT (evm_TU_ctg106_104) promoter:

AGTGTGTATAGCATTTTCATAAGTGTATAGAATAAATAAAGATGATTGGTTTAGAAAAATTAAGTGAGGATATTTTTGATATTTTAAATAATGTACAAAATA
TAATTTAATTTTTTAAAATTAATAGATGAATGCATATAATAATAAAATGTTTAAAATAATTTTTAAAGTACATATAACTCCACCCATTTTAAAATTAAATCAA
ATAAACTATTCACTCTTTTATCTAAAGATGCCTTTATGAACTATTTCAAAGTAATTTAATCTAATTTTAAAATTAATTTATAAGGGAATAAGAATATTAAAAA
GTTGCTGGAAAAAGATGGGTTACAATGAGGGCCCGCCGCCAGCTGTTAAGTCTTCAAAGGAGGCCGGCAGAATTTGACTAATTTTTAGGATAATTCTTT
TTATTAAATTTAAAGATATGTTCAACTTACTGAATTACTAAATTA**ACCTAAC**TTTTAGTCTTAATATTTTAAAATTATTAGTCTTCTTTTTTTCATAAGTTTT
GGTTGGACTAATTCTCAAATTTTAATATAAACTAAATTTGATTGTATCAATAATGAATTTATTTTTAATAATAATTTTTATTTTATTTTATTTAAGATTGAAGG
CTCTATTTGGGACTAAGGGTCTGTATAATATTATTTTTGGGAGGCTTAAAAGTGATTTAAAAAATTTAAAAGTTAATTTTGGT**GTTTGGT**TAAAATAAAA
AATCACTTTTGTCAAAATCAGCCCCTTCCACATCTGATTTTGAAAAGAAATGAATGAGTAACTTTAAATTCTGATTTTGAGAATCAATTTTATCATTTAAT
ATAATTCAATAAATATCCTTGCAATTATTATCTAAACACAAAATTATCCTTATTTAAATTGGAAACAAAATCAATATTTAAAAAAAAAACTAATTGTAATTC
ATCTATTCCTTTCATTTATCCTATTTCTTCTTTATATTCCACATTACATCAAAAGTTCTCTATGGCAATATAGTCTAAATTTTATTTTTAGTTAAAATTATTATA
ATACACAATTAAAATCAATATAGTATAAATTTTATTTTAGTTAAAATTATTATGCTACACAATTAAAAATATAGTATATACATATTTTTATATGTATAAGTAAAT
GTCATCATTCATTATGTACATCTTAGTCATTTGTTTTCTCACATCAGTTTAACAGTAAAATTTATCAAATAAAAATAACTGCTTTTAAAACTCACAATACTT
TTGAAAATAAAATTT**ACCAAAT**ATTTAACTGATTATTTTCACAATTGATTATTTTTATAACACAGCTAACGACAATTATTTTAAAAGTTACAGTATTCCCAA
ACTAACCCTAAGATTGAAATTTTATAAACTGCTTGTTTTATGATTTTCTTCATTTTTTCCATAACTTTTAAAAGCAAATAAAAAGTTATTTTTTTTTATAAAC
AAGTTATTAAATAAGTTACAGCACCTTTTTAAAATTCCTATTACAACCTTATTATTTAAGAAATAGACAATTATAGTTTATTTTTTTCACAGACCTTGATATA
TAAACCATCAGATATCATGTTACTACAACAACTACTATACATTTTCTCAAGTATAAAAATTGATCCAGTAAGTTTATATGTATATTGTATTTTATGTTTATTAT
CATAAGAGTCAATAAGATTTGCATATAAATGTAATGAATACTTTTTTTTTTTGGATGTATGTATTAGTATGATTTTTGTCATATTTTATGAAATATGACAAAA
GGTATGTCATTTAATTAATTTTTTTTCACTTTATGTAATTTTACATATTTTTTTAAAAAAAGTATGTCTAAATAATTTTATTAAATTTTTTTTAAAAAGCAAAA
GTCACTTTAAATTTATATTTATTTAATCATTATATAATAAAACAATACTTTCTTAATAAAATTTACTAAATGTACTTTATTTTTCAAATTTATCGCAACAAATA
CAATTTACTAAAAATATCTTTACTTTTTTTAATCACCCAGCACATGCT

ClCOMT (evm_TU_ctg117_31) promoter:

AACCACTTTCCTCAATGGAGATATAAAGGAAGAGGTTAAACTAAACAATTAGAAGGGCTCATCTTAAGTGAAAATAGTCACTTAATTTGTAAGCATAAG
ATTAAAACAAGCTTATCGTTAGTGGTATTTAAAGTTCCATGACATAATTTCTTC**ATTTGGT**TTCACGGAGAATATTATGGATCAATATATATACCGTGAGGT
CAGTGGAAGAAAAATCATTTTCTTAGTCCTATTTGTATAAGATATTTTGCTTTCTTTAAGTGACTTGGGCTTGTCTCATGGGGTTAAATATTTTCTCTCACA
ACAATTTGATATGAAAGATATGACTAAGACTTCTTATATCATTGGTTTAAAAATTCAAAGAGATAGATCCGGAGGGATATTAGGATTATCTCAAGAAACCT
ATCAATAAAGTTCTTGAGAGATTTCGTATGAAAGATTATTCATCTAATACAGCCTATTATCAAAGGTGACAAATTCAGTGTAAAGCAGTGCCCAGAGAAT
AAAATTCTAAGGGAACAGATGACCAATATTCCTTATGCAGAGTTTAATGTATGCTTTGGGTTGCATAAGCCCTAATATTGCATTTGCAATAAGAATGTTGG
GAAGATATCAATCTAATTCAAGTTTGGAACACAAAAGAACTACAAAGAAAGTATCATTGTACCTTCAAGGAACCAAGGACCACATGCTTACTTATAGAA
GGTCTGATCAATTAGAGGTAATTTGATATACAAACTCTAATTTTGCTAGCTGTTTTGGCACTAAAAGGTCAAATTTATGATATATCTTTTTATTAGCTAGAG
GAGTTATTTCTTGGAGAAGTGCCAAATAGTCATTAATTGCTACCTCAACTACAGAGGTAAAGTTTGTATCTTGTTGTGAACCAACGACATAGGCTAATTG
CCCGAGGAATTTTATTACTAGATTGGGAATTGTTGATTCTATTTCCAGACCACTACGGATCTATTGCGACAATTCAGCTGCAGTTTCTATAGCAAAGAGTA
ACTAAAGTGGAAATTAAAGAAAGCATATCATCATTAAGTACTTAGCTCTTAGAGAGCATATTAGATCACAAATGTGGTTATTGAACATATCAGT**ACCAAA**
TTGATGATTACAGATCCTTTGACGAAGGGATTACCACTGAAGTTGTATAAGGAACATATAGAGCACACGGGACGCAATTTTTTTATATAATTCTATGTAAG
TGAGATACCATGTTTTATGTACTAGTTTTTCAATTGACTCTAAATTTTTAAATTATCAAATTGTCATCTCATATGATTTTTATACATAAGGTTTAAAGAACGA
CGATATGTATAGATATTTGAATAAAATAAAAAAAGGACCATGAATGAGTATTTGGAAGCTTATTCATATAGTATATCCCACATTAAGTACTCACTTTAGGAC
AAGACTATATTGTAATGCATGGAAGGCAATACTTGTTTTTAAAGAACTTGCCACCATGATTCATGGTTTTTCAATTTTTAGTTTGAGTATAAAGTTAAAAC
ATTTAGAGAAAATTAAAGATTATGAGTTTGTAGTGAGGTGGACAAAGTGGGAGAATGTAAGACACGTTTTTGTGTCCTTAAGTGGTAATAATTACC**ATT**
AGGTAATTATGTTTCAGTTAATTTAAACTTTTTGTCCTTATATATATTTATCATCGTTATTGCATTTATTTAATTTAAAATTTTAATATATGAGCAGTTTAAAG
AGAAGTTATATTAACTTCCATTATTCTTAATATCTCTCTACATGTTACAGTGGTTAACAAAATTTAAACGATTATGACCCATCTCTTTCCCTTGCATACATAT
TCTTCTTGATGGGAGAAGAAGTGAACATATTTCAGAAAATAATAAAAATGAAAGCTCCCTAGTTTTACAACTTAATCCTATATATTTCATCTACTCACAAA
AATAGTGGTGTTAGATAGATTGAAAAAATTCAAGTAAAATTATAAAATAAAACTTAGTATCTAAGAGAACCTGATAACT

ClCAD6 (evm_TU_ctg155_195) promoter:

AATGTCTAATATAATATTAGTAACAATCTCTGTATAAAAGAATTAAAAAATAATAATAACTAATAATAAATAAGTCAACACACAGTAAATATGTTTATTAGT
TATGAGATTTCTTTTAGTAGAGCTCCTATAGACTTTGAATTTTATCTTCGCTTTT**ACCTACT**TATGATTTTTAAATTCTATGAAATTCTATTTTTATACATAAT
TGAAGATTTTATCTTTCAGCAGAGTTTAATGATTTTCTCTAGAGTAGAAAAATTGGTGGGTTTTCAAAAAAGCTAATTAAAGAAATTTGGGTTTTTTTTC
TAAAAGAAACCAGTAACAAAACACAATGATTGTTGATAAAAAAAAAAAAAATTAAAGTTAGAAGCTTAAGCAAAGTAAAAATATTGAAGCAGGCTTC**A**
CCAAATGTTTGGGCTTCGGCATAGGCTTCATCGAGTTTTCTGTAGTTAGTCCAAGTTTTTTTTTTTTGGGGTGGGGGGAGGGGACCAATCTCCTCATACT
CATCTCCGATATTTTACGTATGTCCCCGTCCCCTCCCTGTCTCCGTCAGAATTATTTGAGAGAATCCTCATCTCCTCTCCGAATAATAACAGGGGATCTCC
GAGGGTCCCCGGTCCCCGAATAATTAATAGTCTAATACATTTTTTTATTTTTTATTTAAATTAATCATATTAAAATAAAAAATCAGATAAAAATAAAGTTCA
AAATATATCTTACATTAATATTGATCAATTTCAAAGTCACATTTAAAATATAAATTATTAAAATAATTATATTTGTCAATAACTCAATCTTCATAATCCTACAA
TAAAAAGAAAAAAAATATTTTTATGACTGTTGTAAATTATAATTAGGCAGTTCTCTCTTGTATCATTGTGGTTTGGATTAAAAATTTAAGACTTTAATTAGT
TAATTAAGTTTAAAAGTTTAATAATATAAATATTTTGATATTTATTATTTTTAACAATATTTATAATTTTGTTATTTATTTATTAGTAATTTTAAAAATAAAATTA
AAAATTAAAATGGGGAGATGGGTAGGGGATGGAGATTCCACCTCATCCCCGAATAAAAAAATTGGGTAAAAAATTTTCCATGTCCCCTCACCGAATAAAA
AATTGGGTATAAAATTATTCATGTATCCTTCCCGAATAGAAAAAATCCCCAAAAATATCCGTCCCCGTGAGGATATTTGCCATCCCTAGTATTTAGCCAAT
GGTTTCCATATTTTTGTTAATTAAATTGAATCATTCGCTGAAAGTACGTTGCATAATTAATGAATATGAGACATTTCACATGTGAAATGAAATGCTCTATAA
CCAGCCCCAAGTATAAGTGGAAAAATAGACCTTTTTTTTTTCCCCTTGTTTGAATTTTTTTTTTTTGGTCATTTATGTCTTGTCATGAATAATCCCTCAAAA
AGAGTCAACGTAATTAATTTGATCTATTATTTTAACAAGTAAATCAAAATAATATTTTAATTGAAAAGATAATTTGTACTATTTTAATACACGTATTTTTATT
TGTTAAAAAAACAAATTAAGGACAAATTAAATACTCTTATTCAAATTAATAATTTAATTTGCTTTTCAATTCTTTTTTCCCACCCCCAAACTAATGTATATG
ATTTGCTTTGTCTTGAAATTAAGAGAAAAAAGTTTCAGTCAAGTATGCTGCCACAGCCATGTTATGAGCAGTTGCTAAACACCCAGTAGCTTAACTGGG
CAGTGGTGATCGAGTTGAATAGTTGATGGGCAGCCAAAACAAATGCATAGTAAATGACAAAGAAAAATATGTACCAGTAAAAATTTATTTAATAATTGA
CTGGAATGATGTAATTAAACACTTGCTGCGTATTTAGTATTTACTGTGAAAAGTGAAAACAACATTCGCGCACCGTAAACGTTTCCAGCTAATGTATGAC
GTGTATATTCAATTCATTCTAGTTGAGGTTCATTATTTTGCTCTGAATTCAAAGTGACCCATAACAGGTATCAATA

ClCAD9 (evm_TU_ctg115_88) promoter:

TAAATTATTATTTAAATTTTAAATTTATTATATATTATTAATTTTTATTTAATAATTATAATTTTTTTATAATAATTATAATTTAAAAATTACGGTATTTTAATTTC
AAACGAAACCGATAAGTGTTCTGCCGATATTTTTCTTCCAGGCTGCCAGTGTAGGTGAACACATTAAAGACGGATTCCGACAAATTGCCGATATTAATTT
CTGCACGTCATGGGGTCACGCGCTATTGCCGGTTCGCCACCCTATCTCTCAATTAAACGCCTCTTAAATTAGCTGCCGTTGTTACAGTGGTTTATGCTTTA
AAAGCCGCAATTAATCTCTCATCTGTTCAGACCGTCGCCTCCATTCTTCTGCATCTTTTCTACGGACTCGGAGCTTTGACATTTATGATTTATACGAGCTT
TTATATCTTCCTACGTGGTCCCAAGATCTCTCATCGCATTATATATTTTATGTGTTGGTGTGAAATTCAATGTATATGATAATATGAAAAGTGAAAAAGCGC
ATGTATTATGAAATATAATATAAAAACAAAAAACTTCTTTTATACAAAATATGTATATTTAACAAGACAGTTTTGTCCATTTAAACAAAATCTCACGTGTTA
AATTTATTAAAAAAAATTAAATTAAATTTTGGTGCCCAGTATAGGGCTTTTTCTTTTTCCAGAGGTTGCCAAAAGAAGAAACAAATTTATGCCCACAAAT
TAAAGAAGCATCTAGGAATAAAGAGTGGTGTGGGATCTATTCTTGATTGACACACTATCTTTAGAAATTTTACTTTTACTTTATTTTTTTCAAAATTCAGA
AATTATACTTTGATAATGTCAAAAAAATTATTTTTTAAGATTTTGTTGAGCTTATTAATTAACTATTTGATGATATTCAGACCGTAAATATGATATCTTTTGTA
CTAAGGACCCATTTGTAATAATTTTTAGAAGGTTTAAAAGTGATTTTGAAAATTTAAAAGTTAATTTTACTATTTAGTTAAAAAAAAAATCAAAACTACTT
TTGTCAAAATCAACACCTCTTACAGCTGATTTTAAAAAGTAGGAAATGAATAGCTTTTAGATTCTAATTTTGAAAATTAATTTTATTCTTTAATACAATTTC
ATAAATATCCTTAAAATTATTACATAAACCCAAAATTATCATTATTCAAATTAGAAATAAAATTAATATTTTAATAATTTTTTTTATTATAATTCATCAATATAA
ATTTATTAATATCAATTTGTTAGTCGGATTATTATTAAATTTGTTTCACTATAATATAAATTAATTATCAATTGTTTTAAGATGAAAAAATAAAATAAAATCAA
TATATCATAAATTTTATTTTCAGTAAAATTATTACAATACATAATTAAATATCGTGTTTTTATATGTATAAGTAAATTTTATCATACATTATAGGATAAAGCTTC
GATGCAACTGTGGTATCGTGCCACAGTTGCATCCAGCCGTTGGATGCACTTAAATGGGGTTTATTGACATCTAACAGTTGGATACAACTGTGGCACGGT
GCCACAGTTGCACCACAAGTTTTTTCTACATTATATATATTTTATTCATTTATTTTTTCTCAATAGTTTAAAAATAAAATTTATCAAATATTTATAATTATTTTT
AAAACTCACAATATTTTTGAAAACAATAATTATTTTAAAAATTATAATATTTTCAAACTGACCCTAAATCGTTAGACATCTTAGACCGCCAAGTTATATATA
TATATTTTCTACAAATTATTGTAAAAAATTTACAAATAAACTTATAAAAATAATACTTGATAAAAAAAAATTAAGACTTTTTCGGAAAAT**GGTTGGT**TACAG
ATTTCCGTACGGCACTCAAAAACCGTTATATTTAGACGGATTCCAACTTTGGCAACACATTGCCTCCGAAATCACAAGCCAGCTTCTTCTATCATTATTA
CGCACATTTCATTGTTTAAGTGAGAGTGAAGGATAAAGGCCAAAGAGAGAGAGAAAGC

Supplementary Data 2

Cl4CL:

GTCGACGTATATTAACTCAACCAATACTCAAA**ACCAACT**CAGGGAAGCGTATATTAACTCAACCAATACTCAAA**ACCAACT**CAGGGAAGCGTATATTA
ACTCAACCAATACTCAAA**ACCAACT**CAGGGAAGCGAGCTC

ClCCR:

GTCGACTGATGGTAGTGGTGGTTTGTTCGAAA**AGTTGGT**GTTTTGGCCGTGATGGTAGTGGTGGTTTGTTCGAAA**AGTTGGT**GTTTTGGCCGTGATGG
TAGTGGTGGTTTGTTCGAAA**AGTTGGT**GTTTTGGCCGGAGCTC

ClCCoAOMT:

GTCGACGGT**GTTTGGT**TAAAATAAAAAATCACTTTTGTCAAAATCAGCCCCGGT**GTTTGGT**TAAAATAAAAAATCACTTTTGTCAAAATCAGCCCCGG
T**GTTTGGT**TAAAATAAAAAATCACTTTTGTCAAAATCAGCCCCGAGCTC

ClCOMT:

GTCGACGTGGTTATTGAACATATCAGT**ACCAAAT**TGATGATTACAGATCCTTTGACGGTGGTTATTGAACATATCAGT**ACCAAAT**TGATGATTACAGATC
CTTTGACGGTGGTTATTGAACATATCAGT**ACCAAAT**TGATGATTACAGATCCTTTGACGGAGCTC

ClCAD6:

GTCGACGAAGCAGGCTTC**ACCAAAT**GTTTGGGCTTCGGCATAGGCTTCATCGAGGAAGCAGGCTTC**ACCAAAT**GTTTGGGCTTCGGCATAGGCTTCAT
CGAGGAAGCAGGCTTC**ACCAAAT**GTTTGGGCTTCGGCATAGGCTTCATCGAGGAGCTC

ClCAD9:

GTCGACGACTTTTTCGGAAAAT**GGTTGGT**TACAGATTTCCGTACGGCACTCGACTTTTTCGGAAAAT**GGTTGGT**TACAGATTTCCGTACGGCACTCGA
CTTTTTCGGAAAAT**GGTTGGT**TACAGATTTCCGTACGGCACTCGAGCTC

Supplementary Table 1

Gene ID/database	Primer name	Primer sequence (5′–3′)	Purpose/vector
AF176669.1/NCBI	*EGIDH*-qPCR-F	TGGAACAGTCACCCGACACT	RT-qPCR
	EGIDH-qPCR-R	CCTATGTGCAAGCCCTCGTG	
KJ852651.1/NCBI	*GAPC2*-qPCR-F	CCCAGCAAAGACGCTCCAAT	RT-qPCR
	GAPC2-qPCR-R	AGGGGAGCAAGGCAGTTAGT	
evm_TU_ctg48_269/GWH	*ClPAL*-qPCR-F	TTGACCATTGGCCAAGTGAC	RT-qPCR
	ClPAL-qPCR-R	CAGTCCCTTTCATCATGCTG	
evm_TU_ctg113_252/GWH	*ClC4H*-qPCR-F	TTACCGTCTCTAAACTCCGC	RT-qPCR
	ClC4H-qPCR-R	TTCTTGGCTAAGTCGGAGAG	
evm_TU_ctg86_262/GWH	*Cl4CL*-qPCR-F	TCCCCGCCGTCAAGATCAATCC	RT-qPCR
	Cl4CL-qPCR-R	CCATCAACTTGTTGGGCCACGC	
evm_TU_ctg170_30/GWH	*ClCCR*-qPCR-F	AGTGTTCGAGGCCGACGTGTTG	RT-qPCR
	ClCCR-qPCR-R	CAGAATGAGCTCCTTCTCGGGG	
evm_TU_ctg117_31/GWH	*ClCOMT*-qPCR-F	GCGTCCTCAACAAGGGCCAAAG	RT-qPCR
	ClCOMT-qPCR-R	GTGGGCAAGCTCCATGATCAGC	
evm_TU_ctg106_104/GWH	*ClCCoAOMT*-qPCR-F	TGCCATTCCCGACGATGGCAAG	RT-qPCR
	ClCCoAOMT-qPCR-R	AGGAAGAGCAGGGCCTTCTCTG	
evm_TU_ctg155_195/GWH	*ClCAD6*-qPCR-F	GGGATGGGAGCATCACTTACGG	RT-qPCR

	ClCAD6-qPCR-R	ACACCGTGATTCCCGCACACAG	
evm_TU_ctg115_88/GWH	*ClCAD9*-qPCR-F	TGGTTGGGCTGCCAGAGACAAC	RT-qPCR
	ClCAD9-qPCR-R	GGCACAATGCAAGTCCGAGTGG	
evm_TU_ctg70_96/GWH	*ClLaccase*-qPCR-F	CGGGAAGGAGTTACACATAC	RT-qPCR
	ClLaccase-qPCR-R	GAGAACCCATTCTTGGGTAG	
evm_TU_ctg147_35/GWH	*ClPeroxidase*-qPCR-F	GGAGCTACAGCTTTCTCAAC	RT-qPCR
	ClPeroxidase-qPCR-R	GAAGTAAGGAAGCACCCATG	
evm_TU_ctg99_12/GWH	*ClMYB46L*-qPCR-F	GGAGGGAGCTTTAGCAATTAC	RT-qPCR
	ClMYB46L-qPCR-R	GGTTGCACTCAAAGGTCTCG	
evm_TU_ctg46_166/GWH	*ClCYP86B1*-qPCR-F	GTGTGTGGAAAACGATGAGG	RT-qPCR
	ClCYP86B1-qPCR-R	TTGCTTTGCATGGACAGCTC	
evm_TU_ctg47_59/GWH	*ClGPAT5*-qPCR-F	GGGACTCTATTGAAGGATCC	RT-qPCR
	ClGPAT5-qPCR-R	ACCCTAATGCATCCAGCAAC	
evm_TU_ctg65_58/GWH	*ClFAR3*-qPCR-F	ATGGGGCATCTGGATTAGAC	RT-qPCR
	ClFAR3-qPCR-R	GCCCTTTTGATGCCCAAATC	
evm_TU_ctg147_271/GWH	*ClFAR4*-qPCR-F	ACCGGTTGTGATCATTCGAC	RT-qPCR
	ClFAR4-qPCR-R	AAACTCGTTTAGCTGCCCTC	
evm_TU_ctg65_60/GWH	*ClFAR5*-qPCR-F	CAGTCGCACTACGTTTTCAG	RT-qPCR
	ClFAR5-qPCR-R	CTAGAGATATCACCAGGGAC	

evm_TU_ctg74_658/GWH	*ClFeruloyl transferase*-qPCR-F	ACGCCGTTATCGACATGATG	RT-qPCR
	ClFeruloyl transferase-qPCR-R	CCCGCAAGGGGATAATAATG	
JN938957.1/NCBI	*P. digitatum*-qPCR-F	CTTTGCCCCAAGGTGGTCTC	DNA-based qPCR
	P. digitatum-qPCR-R	TGCGGTGGTTGCATCTCTTC	
evm_TU_ctg99_12/GWH	*ClMYB46L*-BamHI-F	GATCTGGTTCCGCGTGGATCCATGGGAAATAATAAACAAGTGAAAGTG	GST fusion/EMSA
	ClMYB46L-NotI-R	TCAGTCAGTCACGATGCGGCCGCTTACTGGTTACATTCAAATGTTTCAAA	
evm_TU_ctg99_12/GWH	*ClMYB46L*-SmaI-F-AD	GGCCAGTGAATTCCACCCGGGATGGGAAATAATAAACAAGTGAAAGTG	pGADT7/Y1H
	ClMYB46L-BamHI-R-AD	CAGCTCGAGCTCGATGGATCCTTACTGGTTACATTCAAATGTTTCAAA	
evm_TU_ctg99_12/GWH	*ClMYB46L*-HindIII-F	GTCGACGGTATCGATAAGCTTATGGGAAATAATAAACAAGTGAAAGTG	pGreenII 62-SK/DLR
	ClMYB46L-BamHI-R-SK	CGCTCTAGAACTAGTGGATCCTTACTGGTTACATTCAAATGTTTCAAA	
evm_TU_ctg86_262/GWH	*Cl4CL*-Pro-SmaI-F	ATCGAATTCCTGCAGCCCGGGGATGATACAACCATAAACTCTTGTATAAAC	pGreenII 0800-LUC/DLR
	Cl4CL-Pro-NotI-R	ATCTCCACCGCGGTGGCGGCCGCTTTTGCAAAGAGGATATGGATATGAG	
evm_TU_ctg170_30/GWH	*ClCCR*-Pro-SmaI-F	ATCGAATTCCTGCAGCCCGGGACTTTTGTATTAATGTCCATCCAATC	pGreenII 0800-LUC/DLR
	ClCCR-Pro-NotI-R	ATCTCCACCGCGGTGGCGGCCGCAAAATGGACTTGAGCGTTCG	
evm_TU_ctg106_104/GWH	*ClCCoAOMT*-Pro-SmaI-F	ATCGAATTCCTGCAGCCCGGGAGTGTGTATAGCATTTTCATAAGTG	pGreenII 0800-LUC/DLR
	ClCCoAOMT-Pro-NotI-R	ATCTCCACCGCGGTGGCGGCCGCAGCATGTGCTGGGTGATTAA	
evm_TU_ctg117_31/GWH	*ClCOMT*-Pro-SmaI-F	ATCGAATTCCTGCAGCCCGGGAACCACTTTCCTCAATGGAG	pGreenII 0800-LUC/DLR
	ClCOMT-Pro-NotI-R	ATCTCCACCGCGGTGGCGGCCGCAGTTATCAGGTTCTCTTAGATACTAAG	
evm_TU_ctg155_195/GWH	*ClCAD6*-Pro-SmaI-F	ATCGAATTCCTGCAGCCCGGGATGAGATTTCTTTTAGTAGAGCTCC	pGreenII 0800-LUC/DLR

	ClCAD6-Pro-NotI-R	ATCTCCACCGCGGTGGCGGCCGCTATTGATACCTGTTATGGGTCAC	
evm_TU_ctg115_88/GWH	*ClCAD9*-Pro-SmaI-F	ATCGAATTCCTGCAGCCCGGGCGGTATTTTAATTTCAAACGAAACC	pGreenII 0800-LUC/DLR
	ClCAD9-Pro-NotI-R	ATCTCCACCGCGGTGGCGGCCGCGCTTTCTCTCTCTCTTTGGC	
evm_TU_ctg99_12/GWH	*ClMYB46L*-SmaI-F-SK	ATCGAATTCCTGCAGCCCGGGATGGGAAATAATAAACAAGTGAAAGTG	pGreenII 62-SK/transient overexpression
	ClMYB46L-BamHI-R-SK	CGCTCTAGAACTAGTGGATCCTTACTGGTTACATTCAAATGTTTCAAA	

Original data for the disease phenotype of lemon fruit under BABA treatment and *P. digitatum* infection

3 dpi

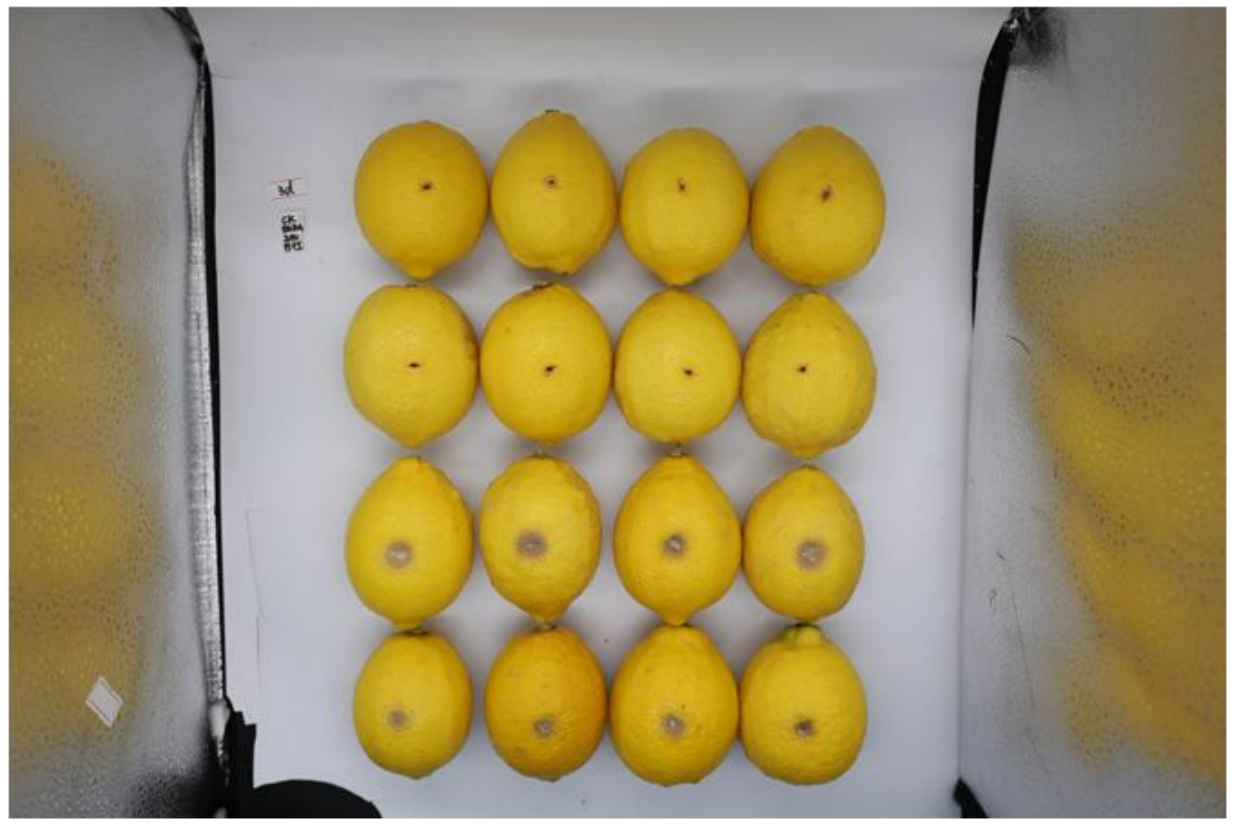

6 dpi

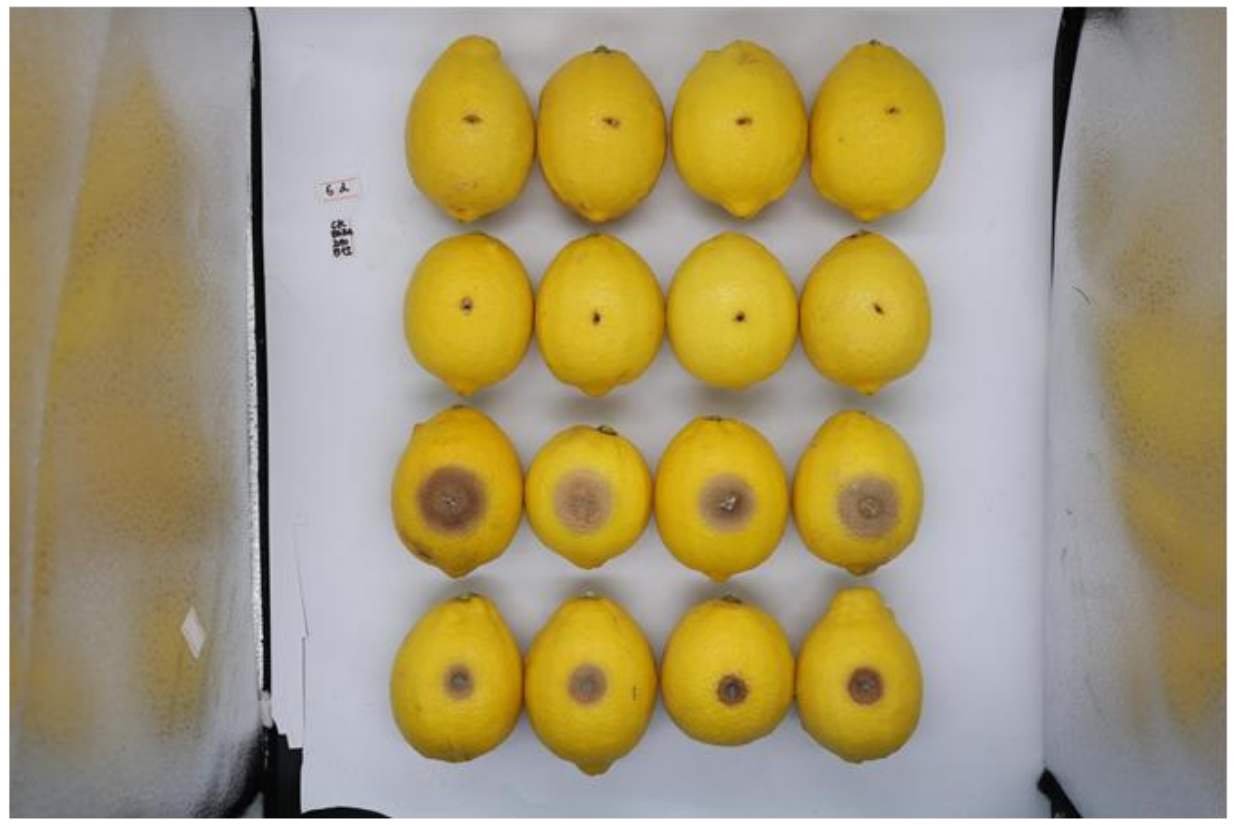

9 dpi

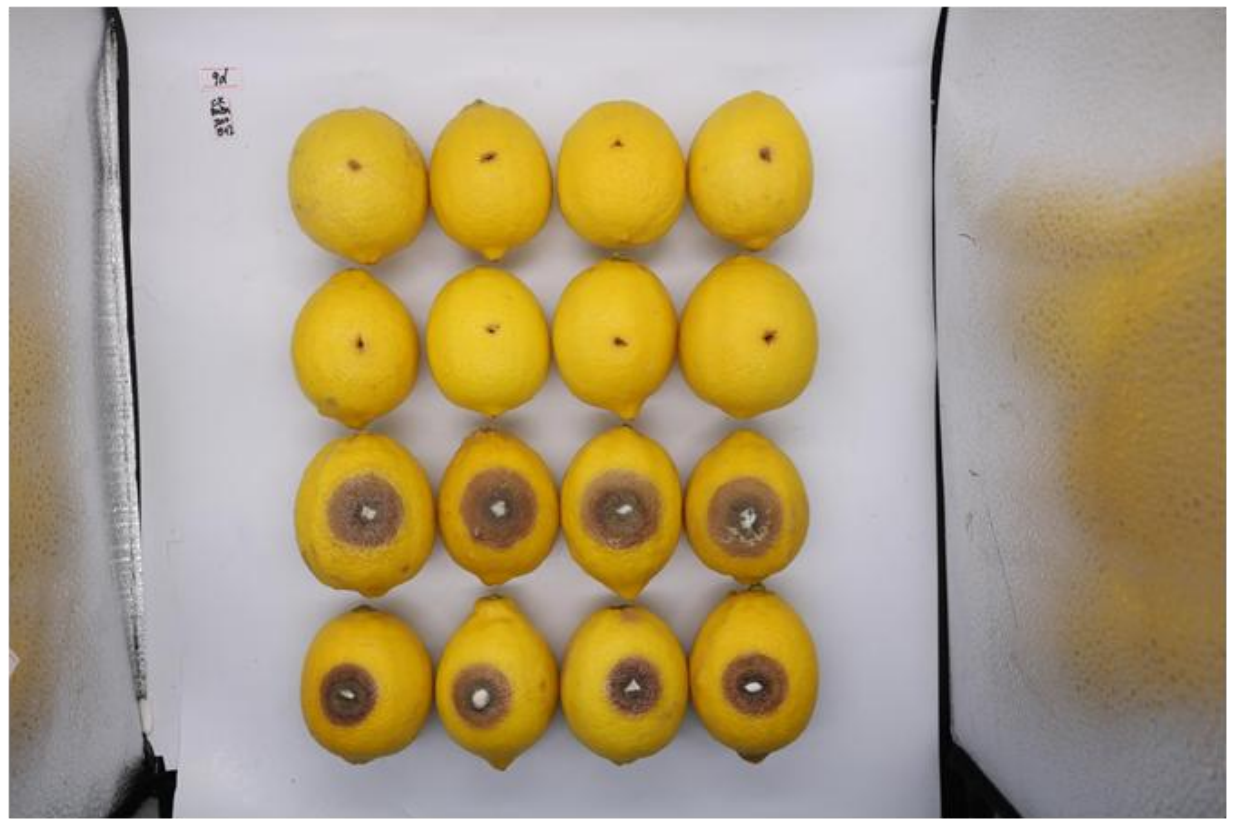

12 dpi

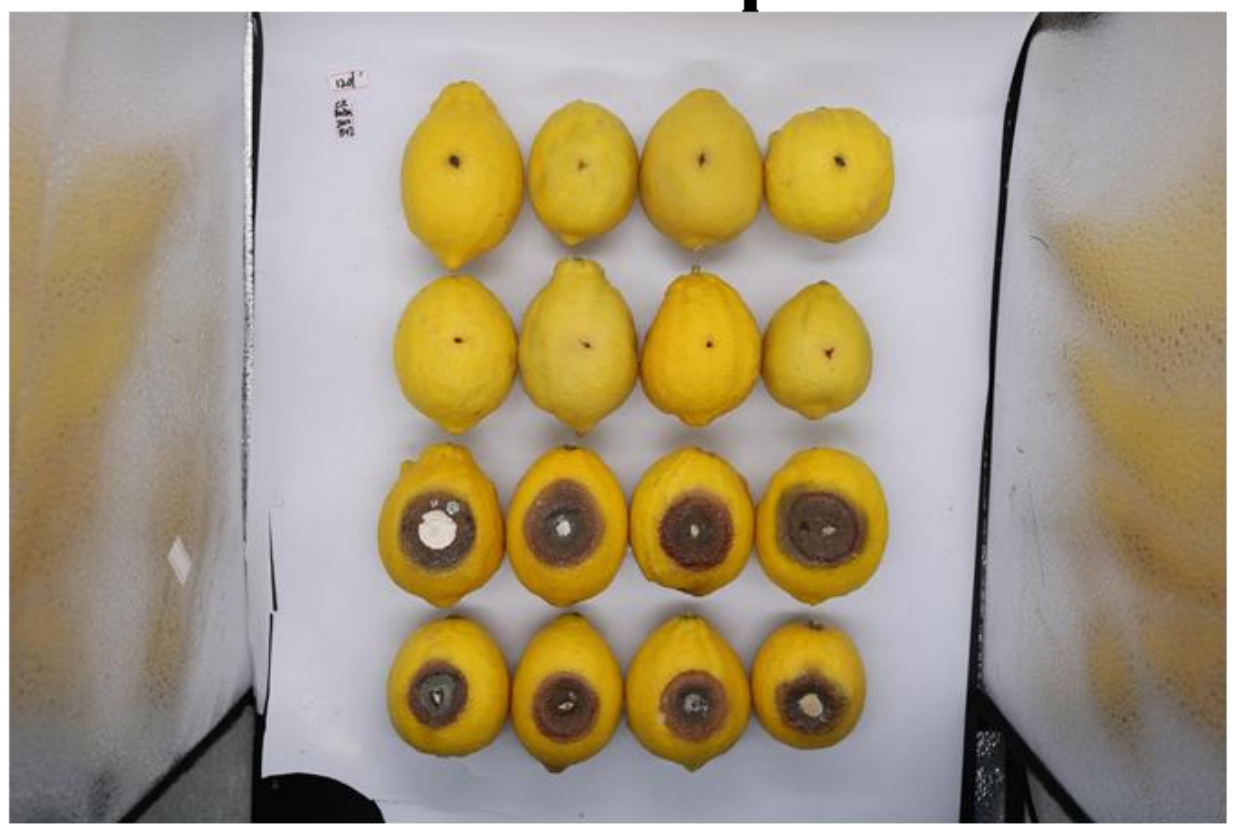

15 dpi

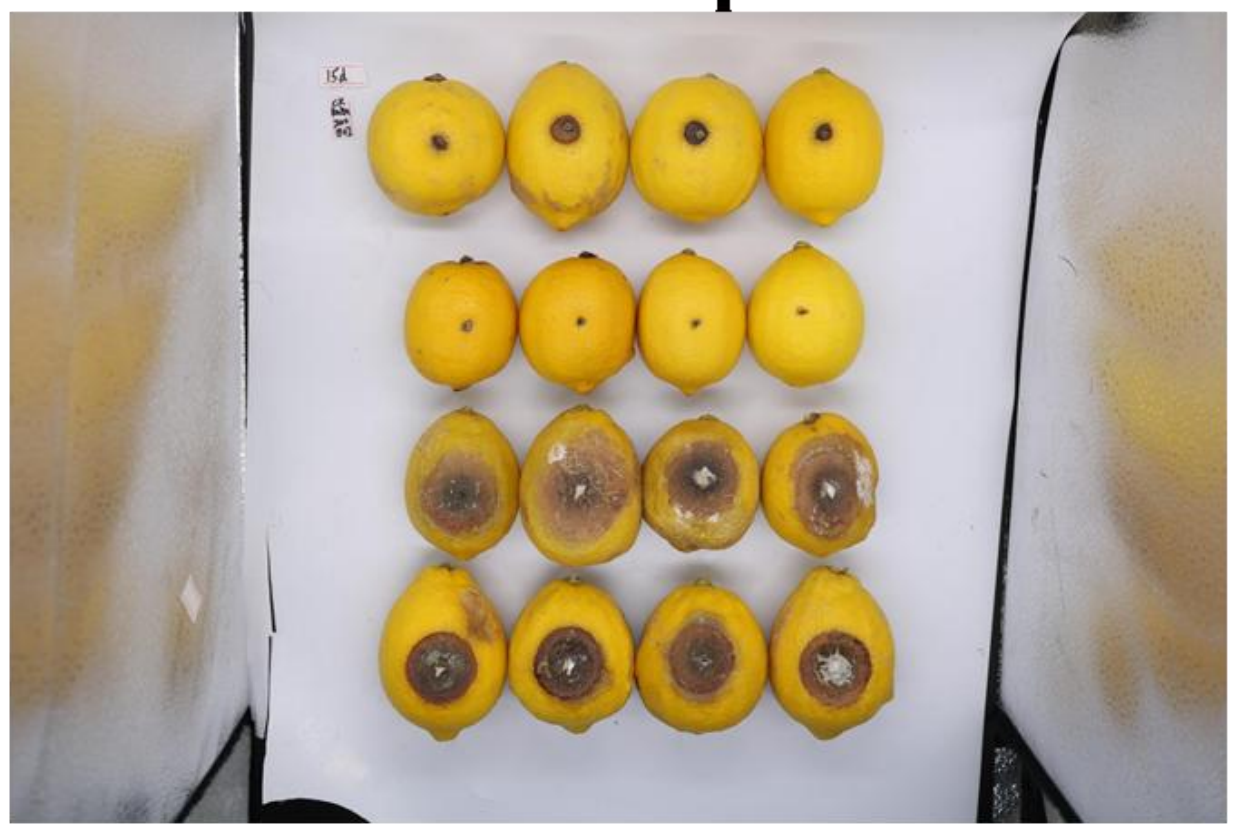

Original data for trypan blue staining of lemon fruit under BABA treatment and *P. digitatum* infection

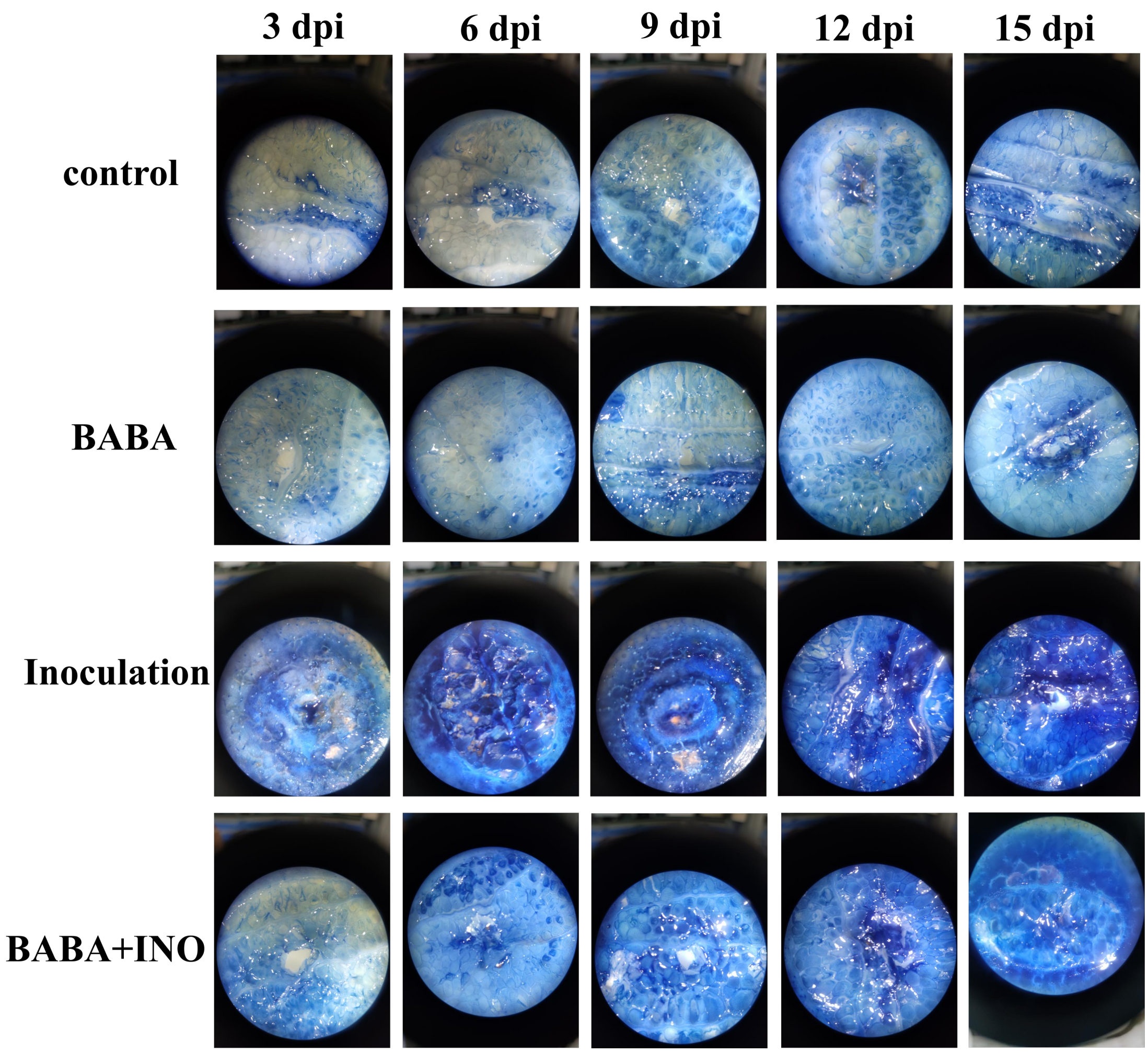

Original images of histochemical lignin staining in lemon fruit following BABA treatment and *P. digitatum* infection

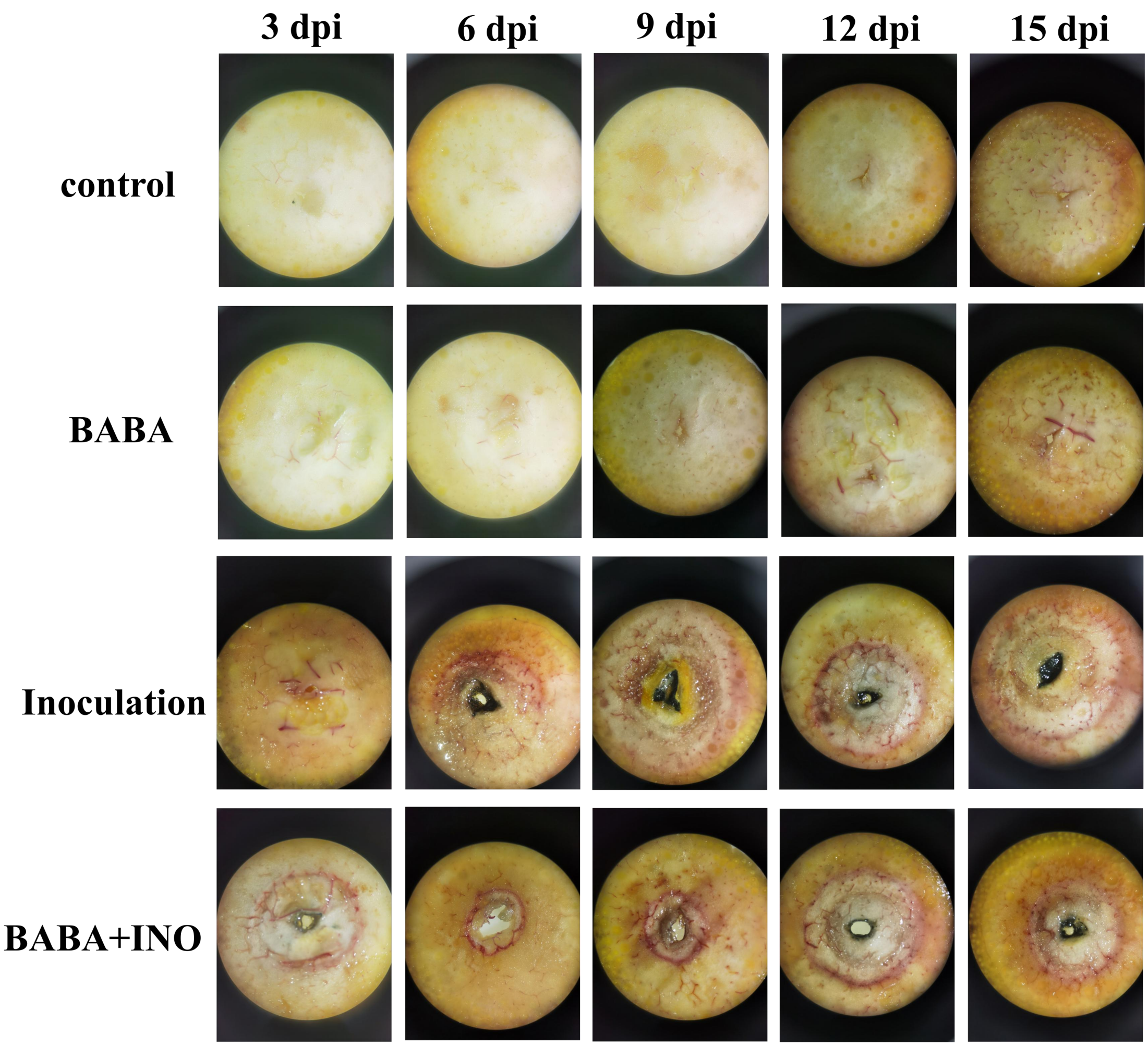

Original data for EMSA

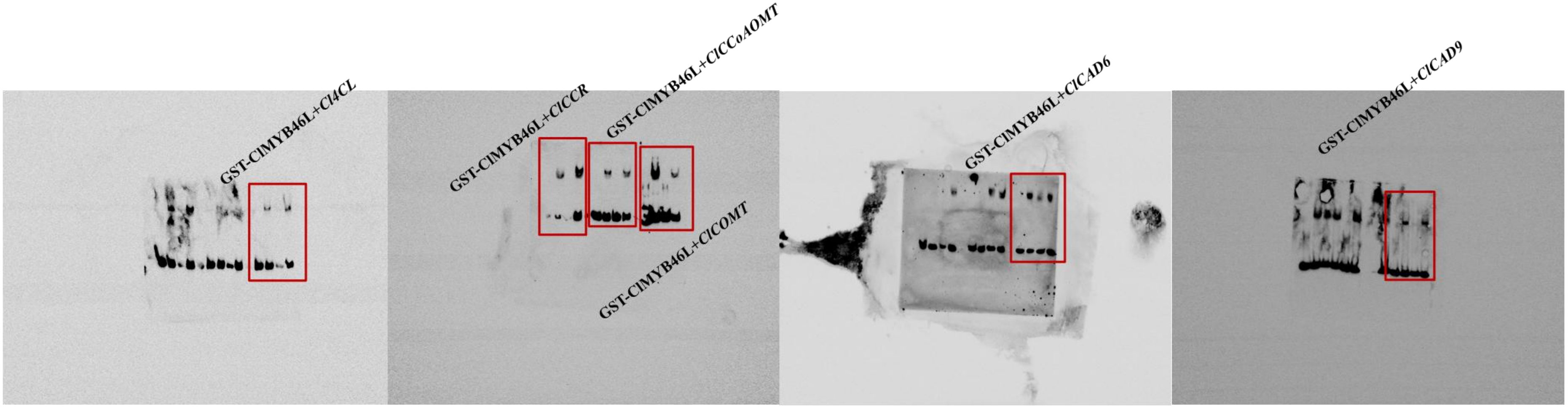

Original Y1H data for ClMYB46L and promoter elements of *Cl4CL*, *ClCCR*, *ClCCoAOMT*, *ClCOMT*, *ClCAD6* and *ClCAD9*

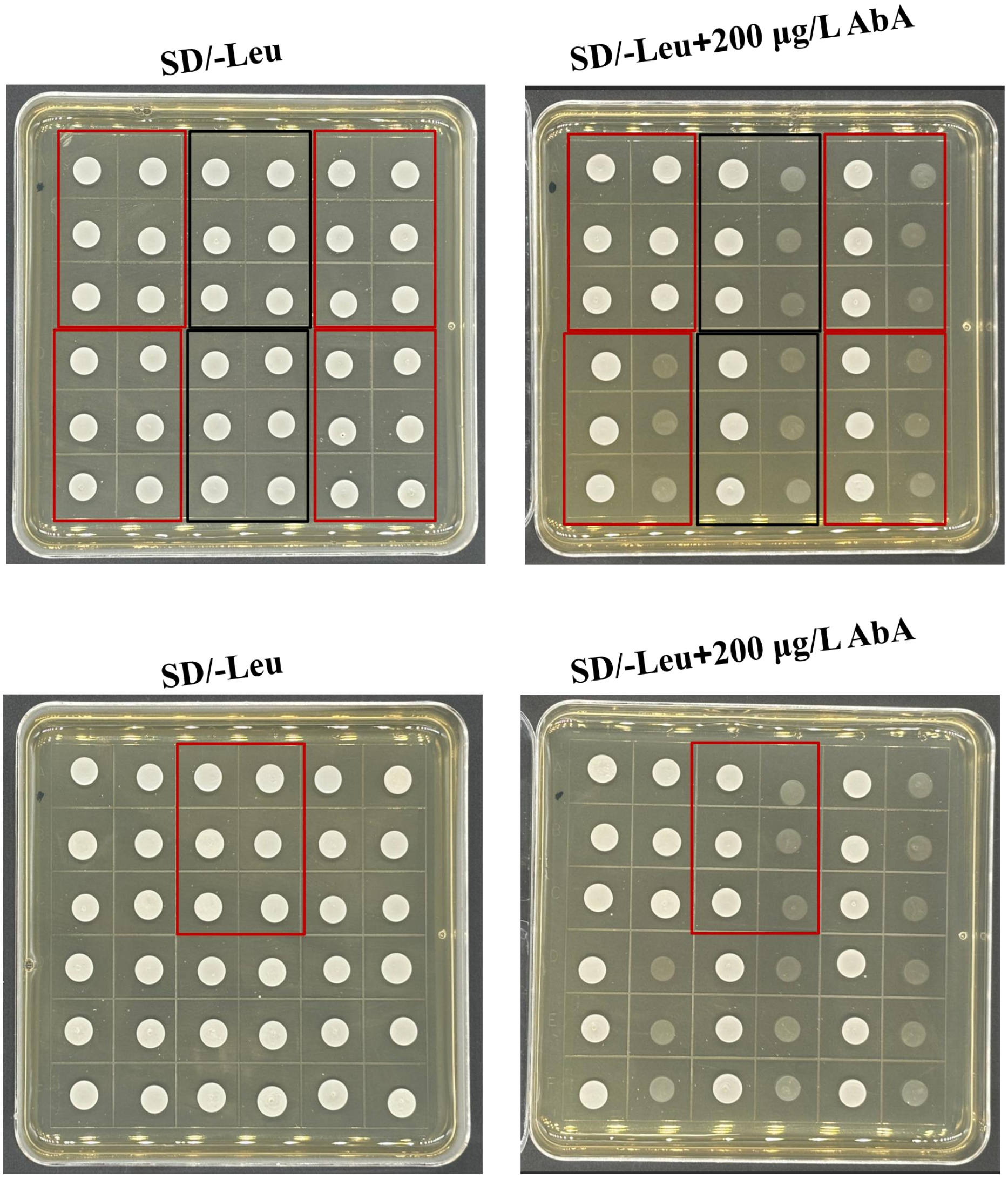

Original data for the disease phenotype of ClMYB46L-overexpressing fruit

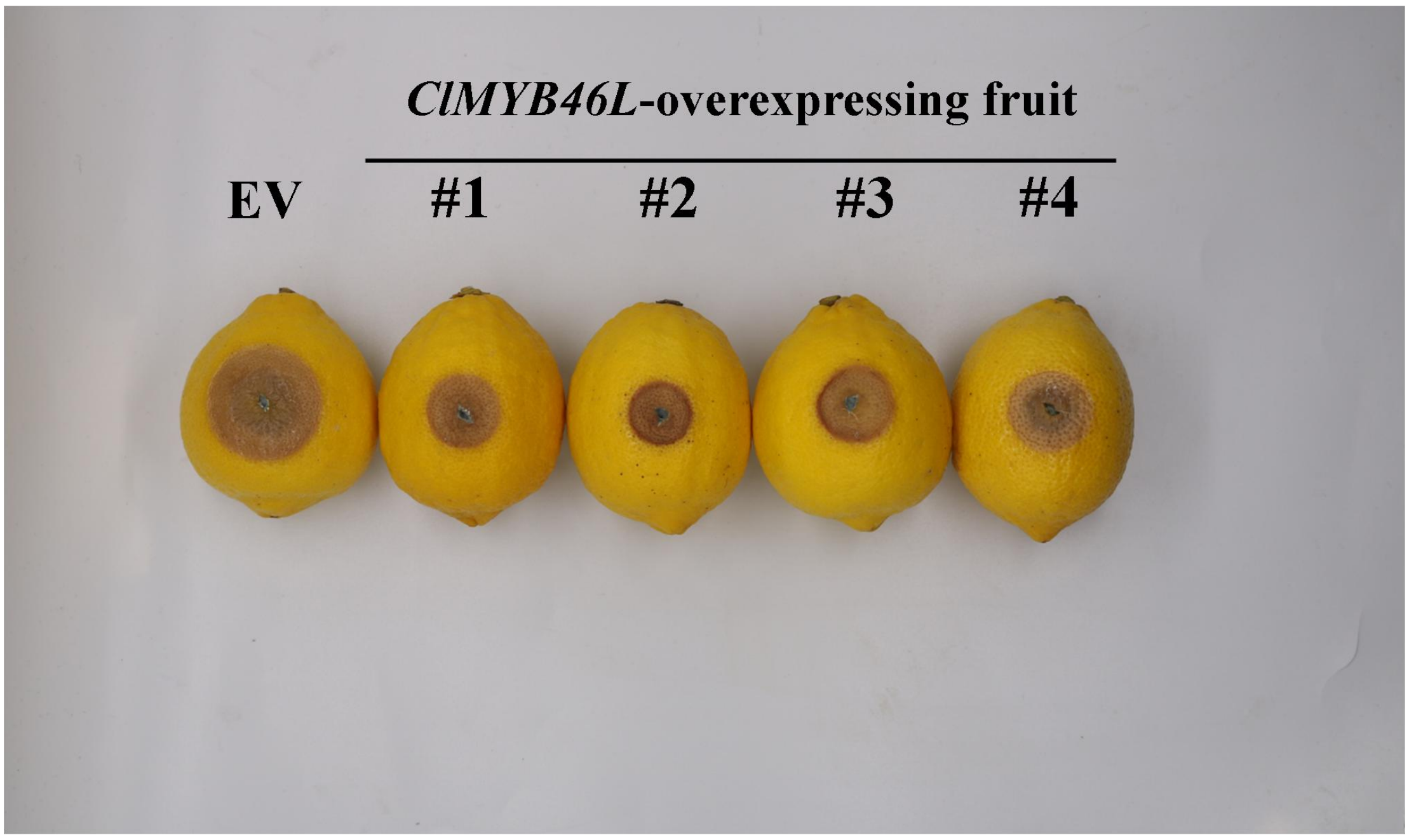

Original data for trypan blue staining of ClMYB46L-overexpressing fruit

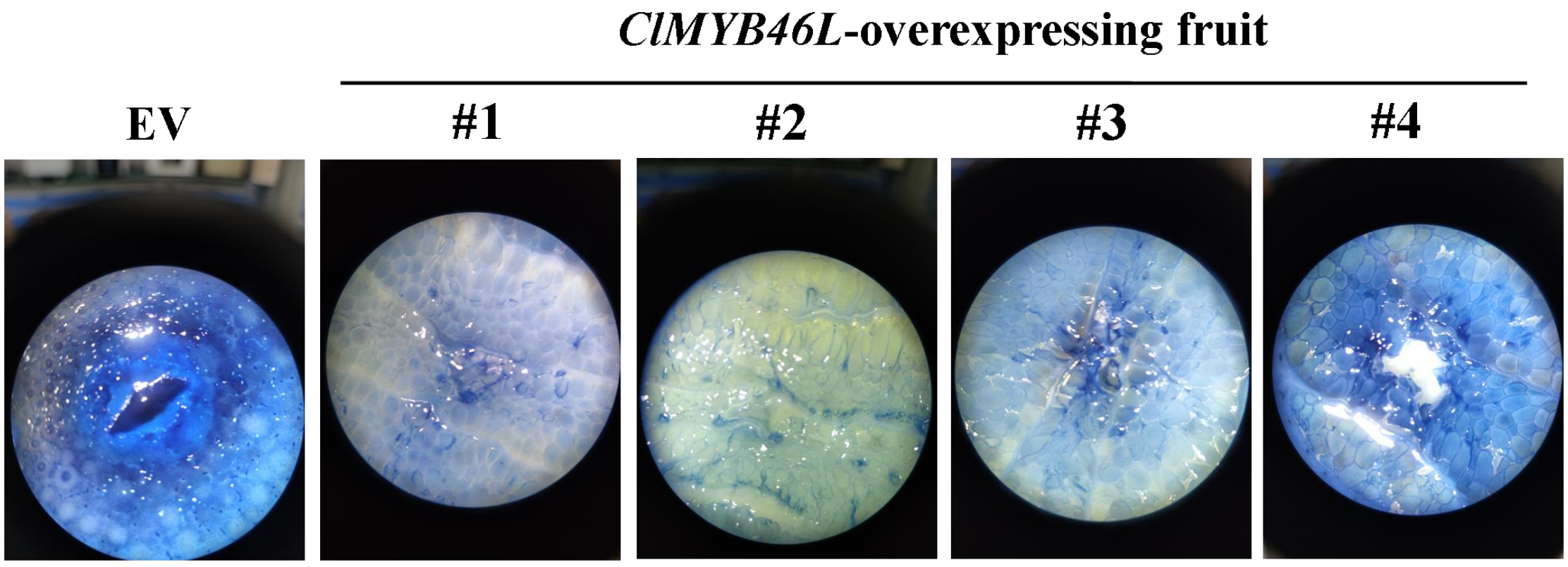

Original data for staining of ClMYB46L-overexpressing fruit

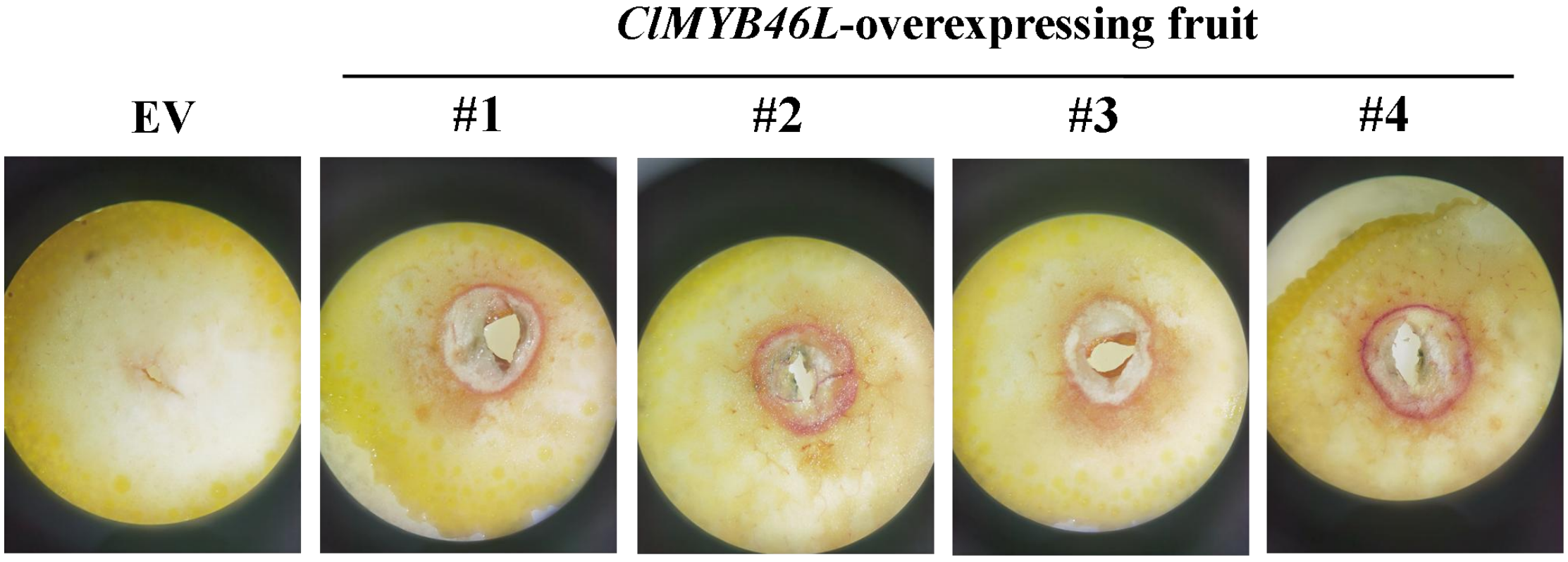

Supplement: Supplementary file 1 [file DataSheet1.pdf]
